# Tuning Proton Transfer Thermodynamics in SARS-CoV-2 Main Protease: Implications for Catalysis and Inhibitor Design

**DOI:** 10.1021/acs.jpclett.1c00425

**Published:** 2021-04-26

**Authors:** Laura Zanetti-Polzi, Micholas Dean Smith, Chris Chipot, James C. Gumbart, Diane L. Lynch, Anna Pavlova, Jeremy C. Smith, Isabella Daidone

**Affiliations:** † Center S3, CNR Institute of Nanoscience, Via Campi 213/A, I-41125 Modena, Italy; ‡ Department of Biochemistry, Molecular and Cellular Biology, The University of Tennessee, Knoxville, 309 Ken and Blaire Mossman Bldg., 1311 Cumberland Avenue, Knoxville, Tennessee 37996, United States; ¶ UMR 7019, Université de Lorraine, Laboratoire International Associé CNRS, 54506 Vandœuvre-lès-Nancy, France; § University of Illinois at Urbana−Champaign, 1110 West Green Street, Urbana, Illinois 61801, United States; ∥ School of Physics, Georgia Institute of Technology, Atlanta Georgia 30332, United States; ⊥ UT/ORNL Center for Molecular Biophysics, Biosciences Division, Oak Ridge National Laboratory, Oak Ridge, Tennessee 37831, United States; # Department of Physical and Chemical Sciences, University of L’Aquila, Via Vetoio, I-67010 L’Aquila, Italy

## Abstract

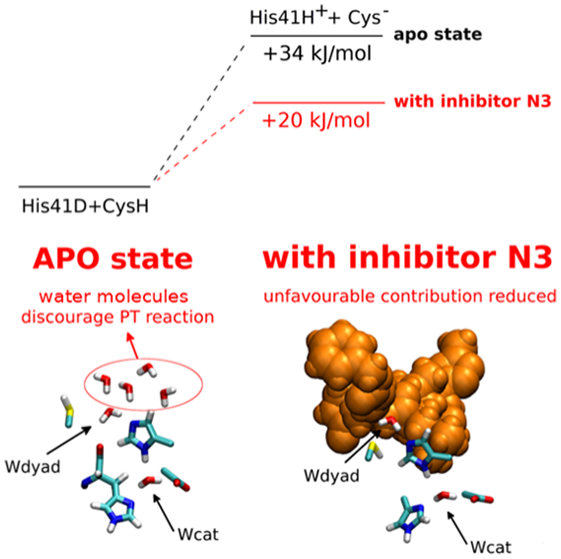

The catalytic reaction in SARS-CoV-2 main protease is activated by a proton transfer (PT) from Cys145 to His41. The same PT is likely also required for the covalent binding of some inhibitors. Here we use a multiscale computational approach to investigate the PT thermodynamics in the apo enzyme and in complex with two potent inhibitors, N3 and the α-ketoamide **13b**. We show that with the inhibitors the free energy cost to reach the charge-separated state of the active-site dyad is lower, with N3 inducing the most significant reduction. We also show that a few key sites (including specific water molecules) significantly enhance or reduce the thermodynamic feasibility of the PT reaction, with selective desolvation of the active site playing a crucial role. The approach presented is a cost-effective procedure to identify the enzyme regions that control the activation of the catalytic reaction and is thus also useful to guide the design of inhibitors.

The rapid and broad spread of the pandemic caused by severe acute respiratory syndrome coronavirus 2 (SARS-CoV-2) has led to an urgent need for effective therapeutics. One of the most promising targets for drug development among coronaviruses is the main protease (M^pro^ or 3CLpro), as this protein plays a key role in viral replication and transcription, cleaving the virus non-structural polyprotein at 11 sites.^1^ Inhibition of its cleavage activity would therefore block the viral replication cycle. In addition, the recognition sequence of SARS-CoV-2 M^pro^ is different from that of all known human proteases, and thus inhibitors of its activity are less likely to be toxic.^2^ Furthermore, the structure of M^pro^ and its catalytic pocket is very similar among the coronavirus family, suggesting that broad-spectrum antiviral drugs might be obtained by targeting this enzyme.^1^

SARS-CoV-2 M^pro^ is a three-domain cysteine protease which is active in its dimeric form^2^ (structural details are given in the Supporting Information, SI). Similar to other cysteine proteases, SARS-CoV-2 M^pro^ features a cysteine-histidine catalytic dyad (Cys145/His41) (see Figure 1). Protein hydrolysis is mediated by the catalytic Cys145 via a nucleophilic attack on the carbonyl carbon of a susceptible peptide bond. It is widely accepted that the imidazole of His41 is the base of the proton transfer (PT) reaction that itself leads to a highly reactive zwitterionic couple (Cys145^–^/His41^+^) which reacts with the substrate.^1^ However, the PT mechanism in the apo enzyme and in the presence of the functional substrate is still under debate.^3−7^

**Figure 1:**
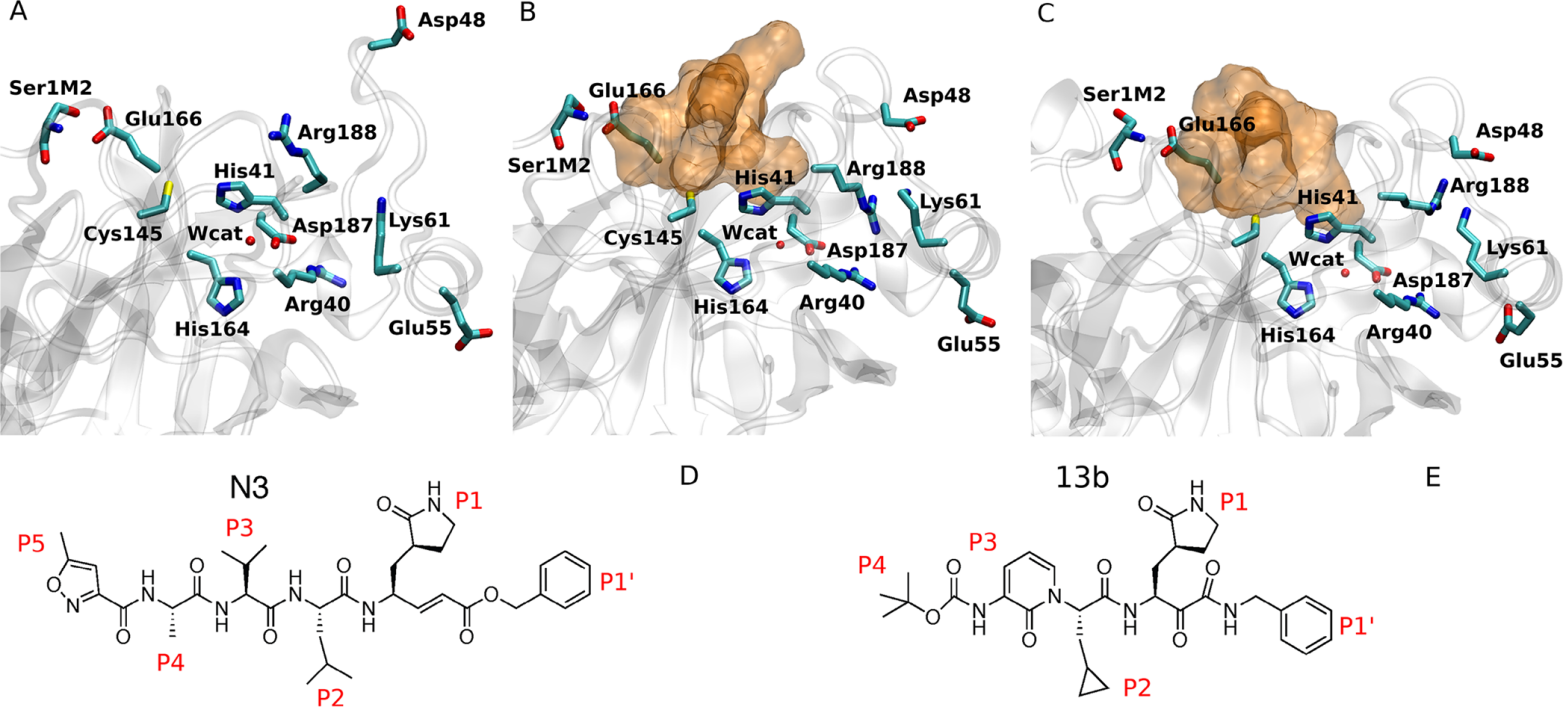
(A–C) Representative structure of the binding site of SARS-CoV-2 M^pro^ and its neighbors in the apo state (A) and in the presence of inhibitors N3 (B) and α-ketoamide **13b** (C). The residues of the catalytic dyad (Cys145 and His41), the catalytic water molecule (Wcat), and some key residues surrounding the binding site are highlighted in licorice. (D, E) Chemical structure of the inhibitors N3 (D) and α-ketoamide **13b** (E).

Some classes of inhibitors of SARS-CoV-2 M^pro^, such as Michael acceptors and ketoamides,^2,8^ also require a deprotonated Cys145 to covalently bind the active site. Diverse binding mechanisms between inhibitors and enzymes featuring an active-site cysteine have been reported, either involving a direct PT reaction between the inhibitor and the cysteine^9^ or requiring a PT reaction from the cysteine to a protein base prior to the binding reaction.^10^ In the case of SARS-CoV-2 M^pro^ previous computational works, wherein Michael acceptors^11,12^ and ketoamides^6,13^ were investigated, suggested that the physiological PT reaction from Cys145 to His41 occurs prior to covalent binding of the inhibitor. These computational works^6,11^ also showed that the PT reaction free energy in the presence of two potent inhibitors of SARS-CoV-2 M^pro^, namely the peptidomimetic N3 and the α-ketoamide **13b**,^2,8^ accounts for 50% and 37%, respectively, of the total activation free energy for the formation of the covalent complex and thus the PT relevantly contributes to the rate-determining step. Hence, the improvement of the stability of the charged catalytic dyad by the inhibitor binding could be a strategy to promote catalysis.^7,11,14^ In this perspective, the knowledge of the protein regions that are responsible for the enhancement or reduction of the thermodynamic feasibility of this PT, can guide the design of the recognition motifs of an inhibitor to the catalytic site of the enzyme with the aim of promoting the PT reaction, and thus the subsequent formation of a covalent bond.

The present work focuses on the investigation of the thermodynamics of the protonation states of the catalytic dyad and of the PT reaction in SARS-CoV-2 M^pro^, both in the apo enzyme and in complex with the inhibitors N3 and **13b**. These two inhibitors are peptidomimetic compounds shown in Figure 1 D,E. N3 features a γ-lactam group at position P1, a benzyl ester moiety at position P1′, leucine, valine, and alanine side chains at positions P2, P3, and P4, respectively, and an isoxazole group at position P5.^8^**13b** is a capped dipeptide with an α-ketoamide warhead, a γ-lactam moiety designed as a glutamine surrogate at position P1, a benzyl group at position P1′, a cyclopropyl methyl moiety at position P2, a pyridone in the P3–P2 position, and a hydrophobic Boc group at position P3.^2^

To investigate the thermodynamics of the PT reaction, we use a hybrid quantum/classical approach for the investigation of chemical processes in complex systems based on the joint use of classical molecular dynamics (MD) simulations, quantum mechanical (QM) calculations, and the perturbed matrix method (PMM).^15^ The MD-PMM approach has been successfully used to investigate the thermodynamics of electron transfer and PT reactions^16−23^ and to identify the protein/enzyme sites that are able to modulate the charge-transfer energetics,^19,23^ also providing hints for new possible mutations. With the same approach, we focus here on the identification of the enzyme regions that can be targeted to inhibit its catalytic activity with particular attention to the role played by the solvent.

As commonly done in hybrid multiscale approaches,^24−27^ also for the investigation of enzyme catalysis,^3,5,12,28−31^ the portion of the system in which the chemical event takes place is treated quantum mechanically (the quantum center, QC) while the rest of the system is treated classically and atomistically and exerts an electrostatic perturbation on the QC electronic states. However, the main difference with other hybrid methods is that in the MD-PMM the whole system configurational space (including the QC) is sampled by fully classical MD simulations and the electrostatic perturbation of the environment on the electronic properties of the QC is included *a posteriori*. To include the perturbation of the environment on the QC quantum states, the electrostatic potential and electric field that each atom of the environment exerts on the QC are evaluated at each frame of the MD simulation, and their effects on the QC quantum properties (e.g., energies) are calculated.

Therefore, the instantaneous quantum properties of the QC in the presence of the environment (i.e., the perturbed properties) are obtained by expanding the perturbation operator instead of using full electronic structure calculations. This approximation allows us to estimate the perturbed quantum properties for all the configurations obtained from classical MD simulations of the whole system (including the quantum part) and to employ extensive sampling for the calculation of the reaction thermodynamics. This extensive sampling also allows us to investigate the coupling of the quantum observables with the classical degrees of freedom of the environment, giving insights into the molecular determinants affecting the free-energy change. When charge-transfer reactions are investigated, the donor and acceptor can be treated either as two separate QCs (when they can be considered electronically decoupled) or as a single QC. In the present case, the donor and acceptor are treated as a single QC when close together and hydrogen bonded (see below). It has to be pointed out that the accuracy of the overall approach relies on the accuracy of both the QM calculations and the force field employed for the classical MD simulations. More details on the MD-PMM approach can be found in the Methods section and in the SI.

To investigate the active site PT reaction in SARS-CoV-2 M^pro^, we select the side chains of Cys145 and His41 as QCs. For the configurations in which a direct hydrogen bond (HB) between the catalytic dyad residues is present (i.e., if the distance between the sulfur atom of Cys145 and the ε nitrogen of His41 is less than 0.35 nm and the corresponding HB angle is less than 35°), Cys145 and His41 are treated as a unique QC, while when the HB is not present they are treated as separate QCs (see the SI for details on the definition of the QCs). QM calculations are performed on the above-defined QCs in both the reactant and product ensembles as defined by the following PT reaction:
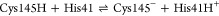
1

Three MD simulations were performed (details on the MD simulations can be found in the SI). Two of these simulations featured both Cys145 and His41 in their neutral state (the reactant ensemble, Cys145H + His41): one with His41 protonated at N_ε_ (His41E) and one with His41 protonated at N_δ_ (His41D). In its neutral state, His41 can be protonated either at its ε or at its δ nitrogen, possibly leading to a different structural and dynamical behavior of the catalytic site and its neighborhood. The third MD simulation was carried out with Cys145 and His41 forming an ionic couple (the product ensemble, Cys145^–^ + His41H^+^). Information on the catalytic dyad configurations and interactions is provided in the SI, and a more extensive structural analysis of the MD simulations can be found in our previous work.^32^ Then, by applying the MD-PMM approach, we computed at each MD frame for each simulation ensemble the time evolution of the energy change upon PT that provides the reaction free energy Δ*G*^0^ (see eq 6 in the SI).

The computed PT reaction free energies reported in Table 1 clearly show that in the apo state both residues of the catalytic dyad are neutral. The zwitterionic couple is in fact at a higher energy for both His41E and His41D in the reactant state (by 34 and 37 kJ/mol for His41D and His41E, respectively; see Table 1). Previous findings obtained with a different computational approach indicate, in agreement with the present results, that the zwitterionic couple lies at a higher energy.^5,6^ However, the free energy cost for the formation of the ionic couple reported in these earlier studies is ∼12 kJ/mol, i.e., lower than the present one by 22 and 25 kJ/mol considering the His41D and His41E states, respectively. We cannot rule out that the approximations of the MD-PMM approach, such as inaccuracies in the QM calculations, can affect the PT free energy. As a matter of fact, QM calculations involving thiol/thiolate reactivity have proven to be quite challenging, and some limitations of common functionals have been pointed out.^33,34^ In addition, it was recently suggested,^35^ on the basis of constant-pH MD simulations, that Cys44 can be deprotonated in the apo state of SARS-CoV-2 M^pro^. As this residue is located only ∼6.5 Å away from His41, in its deprotonated state Cys44 could relevantly affect the PT energy, favoring the dyad charge separated state. This would provide a lower PT free energy compared to the one computed here using protonated Cys44.

**Table 1:** Calculated Free Energy Difference (Δ*G*^0^, kJ/mol) for the Proton Transfer Reaction in Eq 1 in the Apo State and in the Presence of Inhibitors N3 and **13b** and for the Tautomerization Reaction of His41 in the Apo State^a^

	Δ*G*^0^
(Cys145H + His41E ⇌ Cys145^–^ + His41H^+^)_apo_	37
(Cys145H + His41D ⇌ Cys145^–^ + His41H^+^)_apo_	34
(His41E ⇌ His41D)_apo_	3
(Cys145H + His41D ⇌ Cys145^–^ + His41H^+^)_N3_	20
(Cys145H + His41E ⇌ Cys145^–^ + His41H^+^)**_13b_**	31

a The standard error for the computed values is ∼6 kJ/mol and is obtained using three sub-trajectories for each MD simulation. Details on the Δ*G*^0^ calculation can be found in the SI.

The above results also show that the His41E and His41D reactant states are essentially isoenergetic, with His41E slightly more stable with respect to His41D (by ∼3 kJ/mol). This small free energy difference upon tautomerization suggests that in the apo state at physiological temperatures both protonation states may be accessible. This agrees well with a previous computational work in which the role of His41 and its protonation state was addressed.^13^

In order to compute the energy variation upon PT in the presence of the N3 and **13b** inhibitors inside the substrate binding pocket, we simulate the complex between the non-covalently bound inhibitors and the enzyme. A recent computational work addressing the role of histidine protonation in influencing the structural stability of SARS-CoV-2 M^pro^^32^ showed that in the presence of the inhibitor N3 the most stable state is the one with His41D, while in the presence of **13b** the most stable state is the His41E one. Therefore, the computation of the PT energy in the presence of N3 and **13b** focuses on the His41D and His41E reactant states, respectively. As for the apo-enzyme, for each inhibitor two MD simulations are performed for the non-covalent complex, i.e., one in the reactant ensemble and one in the product ensemble.

From the MD-PMM calculations we obtain that, in the presence of the inhibitor N3 in the active-site, the PT reaction free energy is significantly lower, changing from Δ*G*^0^ = 34 ± 6 kJ/mol to Δ*G*^0^ = 20 ± 6 kJ/mol (see Table 1 and Figure 2). The positive free energy difference upon PT estimated here is in agreement with previous recent computational works^11,12^ showing that in the presence of N3 the ionic couple is at a higher energy with respect to the reactant state. In these previous calculations free-energy changes upon PT of ∼5.5 kJ/mol^12^ and ∼43 kJ/mol^11^ were reported. Our estimate of the PT free energy in the presence of N3, i.e., 20 kJ/mol, lies approximately halfway between the two previously calculated values. The differences between the three estimates may arise from the different levels of the QM calculations used and different sampling methods, as previously pointed out.^12^ Other differences in the details of the force fields employed, such as the protonation states of the residues in the vicinity of the active site and the modeling of the non-covalent complex, can also possibly lead to different free-energy estimates.

**Figure 2:**
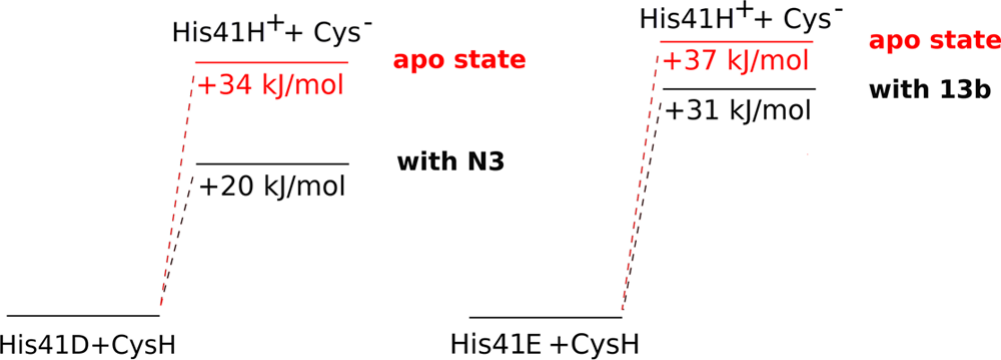
Representation of the free energy change upon PT in the apo state and in the presence of inhibitors N3 and **13b**.

Our calculations show that the zwitterionic couple is at a lower free energy in the presence of the inhibitor N3 than in the apo state (see Figure 2). While this is in contrast with the results of Ramos et al.,^11^ a reduction of the free energy cost in forming the ionic couple was shown for SARS-CoV in the presence of the functional substrate.^36^ We believe that the decrease of the PT free energy in the presence of N3 is consistent with the experimental observation of a very fast inactivation-rate constant for covalent bond formation, that is also determined by the PT reaction step.^8^ As a matter of fact, the inability to efficiently promote the PT reaction has been suggested to contribute to the low inhibition potencies of known inhibitors.^7,36^

The calculation of the free energy change upon PT in the presence of **13b** shows a modest reduction of the PT reaction free energy with respect to the apo state: Δ*G*^0^ = 37 ± 6 kJ/mol in the His41E apo state and Δ*G*^0^ = 31 ± 6 kJ/mol in the His41E state in the presence of **13b** (see Table 1 and Figure 2). The computed value for the free energy difference upon PT is in good agreement with a previous computational estimate for the **13b**-SARS-CoV-2 M^pro^ non-covalent complex (∼30 kJ/mol).^6^

To gain insights into the molecular mechanisms that tune the energetics of the catalytic site in the reactant state, we analyzed the electrostatic potential contribution to the PT energy in the apo and inhibitor-bound states. In fact, the most important contribution to the PT energy fluctuations around the unperturbed ground state energy difference along the MD simulations is given by the electrostatic potential felt by the QCs^37^ (see The Perturbed Matrix Method section in the SI). We therefore analyzed the contribution of each protein residue to the electrostatic potential in order to understand which protein regions contribute the most to the PT energy. This analysis reveals which are the enzyme sites that control, via a conformational electrostatic effect, the catalytic activity that is triggered by the PT reaction in the binding site.

In what follows we report the results obtained for the apo state with His41D and the changes induced by the presence of N3, while the results for the apo state with His41E and the changes occurring upon binding of **13b** are relegated to the SI, as the case of N3 is more informative on the protein and solvent contributions to the PT energy change upon binding. This is because the lowering of the free energy cost in forming the ionic couple is larger upon binding to N3 (from 34 kJ/mol in the apo state to 20 kJ/mol in the bound state) than to **13b** (from 37 kJ/mol in the apo state to 31 kJ/mol in the bound state).

In Figure 3A we report *qV*, i.e., the contribution to the PT energy due to the electrostatic potential computed in the QCs center of mass, of each residue in the MD simulation of the apo state with His41D. In the figure the residues with a negative contribution exert an electrostatic effect that favors the PT reaction, while the opposite is true for the residues with a positive contribution. The residues that most relevantly contribute to the PT energy are Arg40, Glu166, and Arg188 (positive contributions disfavoring PT) and Asp187 (negative contribution favoring PT). These are charged residues in the vicinity of the catalytic dyad that, besides being energetically relevant, are also structurally relevant (see SI for the details). The contribution of Ser1 of the other monomer, related to the one of Glu166 (see SI for the details), can also be seen.

**Figure 3:**
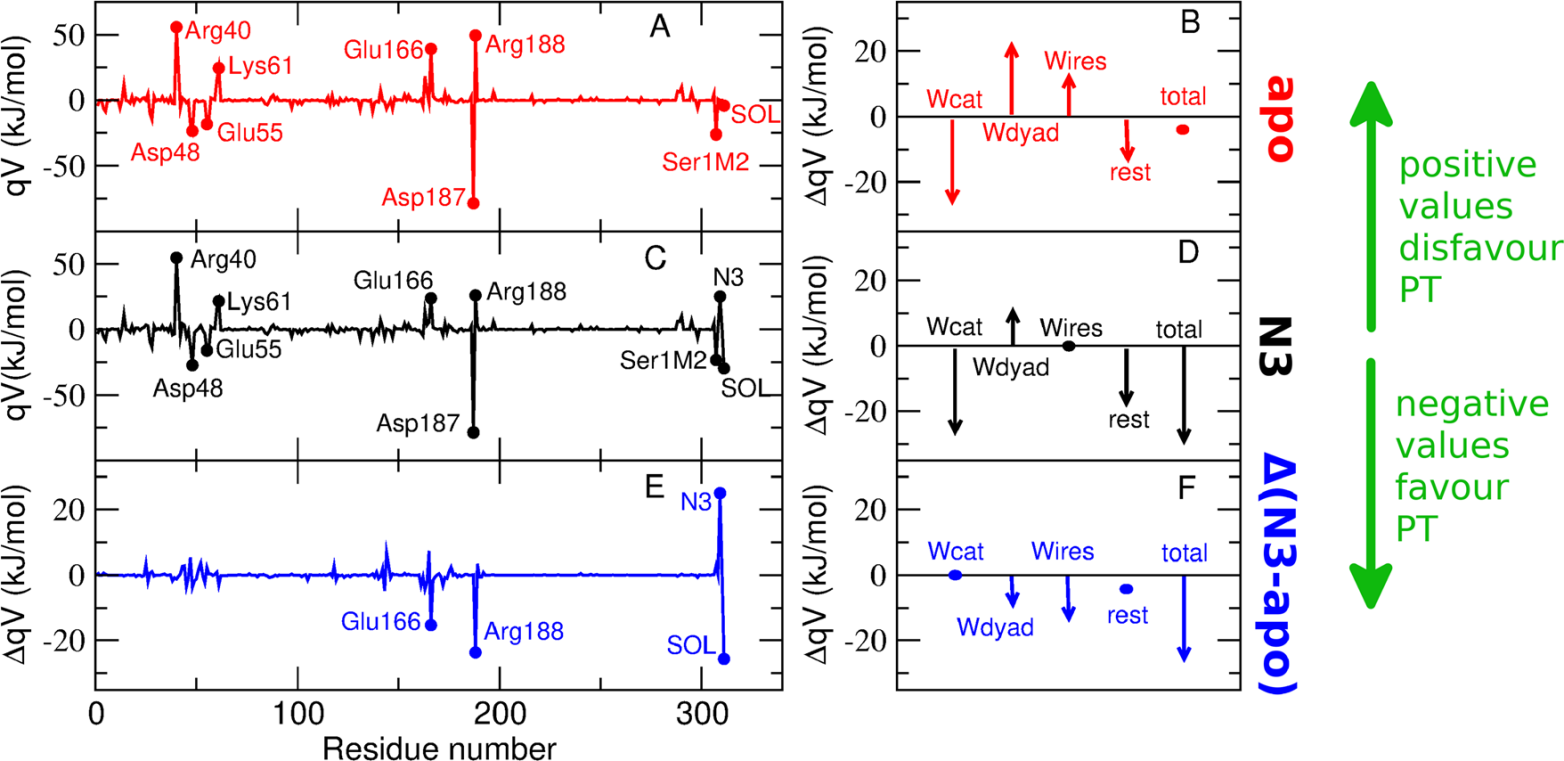
(A, C) *qV* is plotted for each protein residue and all the water molecules as an additional virtual solvent residue (SOL) for the apo state (A) and in the presence of the inhibitor N3 (C). *qV* is the mean value along the MD trajectories of the contribution due to the electrostatic potential to the PT energy. The residues featuring an absolute value of *qV* higher than 20 kJ/mol are labeled in the figure. The residues with a negative contribution exert an electrostatic effect that favors the PT reaction, while the opposite is true for the residues with a positive contribution. The contribution of the solvent in the apo state is labeled for the sake of comparison between the panels, but is approximately null. The contributions of the residues of the catalytic dyad (His41 and Cys145) are not included in the plot. The contributions of the residues of the second monomer (except Ser1M2) are not shown, being negligible. (E) Δ(*qV*) = *qV*(N3) – *qV*(apo) is plotted for each protein residue and SOL. The residues featuring an absolute value of qV higher than 10 kJ/mol are labeled. The contributions of the residues of the catalytic dyad (His41 and Cys145) are not included in the plot. The residues with a negative contribution are those that contribute to lower the PT energy in the presence of the inhibitor with respect to the apo state while the opposite is true for the residues with a positive contribution. (B, D, and F) Dissection of the contribution of the solvent (SOL); the contributions of Wcat, Wdyad, the molecules forming the wires, and the rest of the water molecules are reported together with the total solvent contribution for the apo state (B), in the presence of N3 (D), and for the difference Δ(*qV*) = *qV*(N3) – *qV*(apo) (F).

Three additional minor contributions can also be observed in Figure 3A: Asp48, Glu55, and Lys61. The contribution of these charged residues, which are located on the protein surface in the His41 loop not far from the catalytic dyad cleft, shows that the catalytic activity might be modulated by allosteric control by acting on regions outside the active site cleft. In the attractive framework of allosteric inhibition,^38,39^ a possible inhibition strategy could be to target one of the identified regions in order to promote an allosteric modulation of the PT energetics.

Concerning the contribution of the solvent it can be seen from Figure 3A that the water molecules, considered collectively, do not contribute to the PT energy change in the apo state. However, the analysis of the separate contributions arising from the water molecules closest to the catalytic dyad, reveals interesting features (see Figure 3B). In the crystal structure of SARS-CoV-2 M^pro^ (PDB 6wqf) a highly buried water molecule is present, which is packed in a tight HB network involving His41 N_δ_, His41 backbone NH group, the side chain oxygens of Asp187 and His164 N_δ_ (see Figure 4). This HB network is conserved in other available crystal structures (e.g., PDBs 6y2e, 7bqy, 6y2g, 6yb7), and the buried water molecule, Wcat, has been suggested to play an active role in the catalytic PT reaction.^7,40^ The HB network observed in the aforementioned crystal structures is well conserved in the MD simulation of the apo state with His41D (see SI, Figure S9). Another well-conserved water molecule, Wdyad, can be found in several crystal structures (see, for example, PDBs 6wqf and 6y2e), located within the catalytic dyad, almost bridging the sulfur atom of Cys145 and the N_ε_ atom of His41 (see Figure 4) and is preserved and hydrogen bonded with His41 N_ε_ in the MD simulations with occupancies of ∼80% and ∼55% in the apo state and in the presence of N3, respectively. This water molecule was suggested to play a role in the proton transfer from His41 to the inhibitor after the formation of the covalent bond between the inhibitor and the thiolate of Cys145.^11^

**Figure 4:**
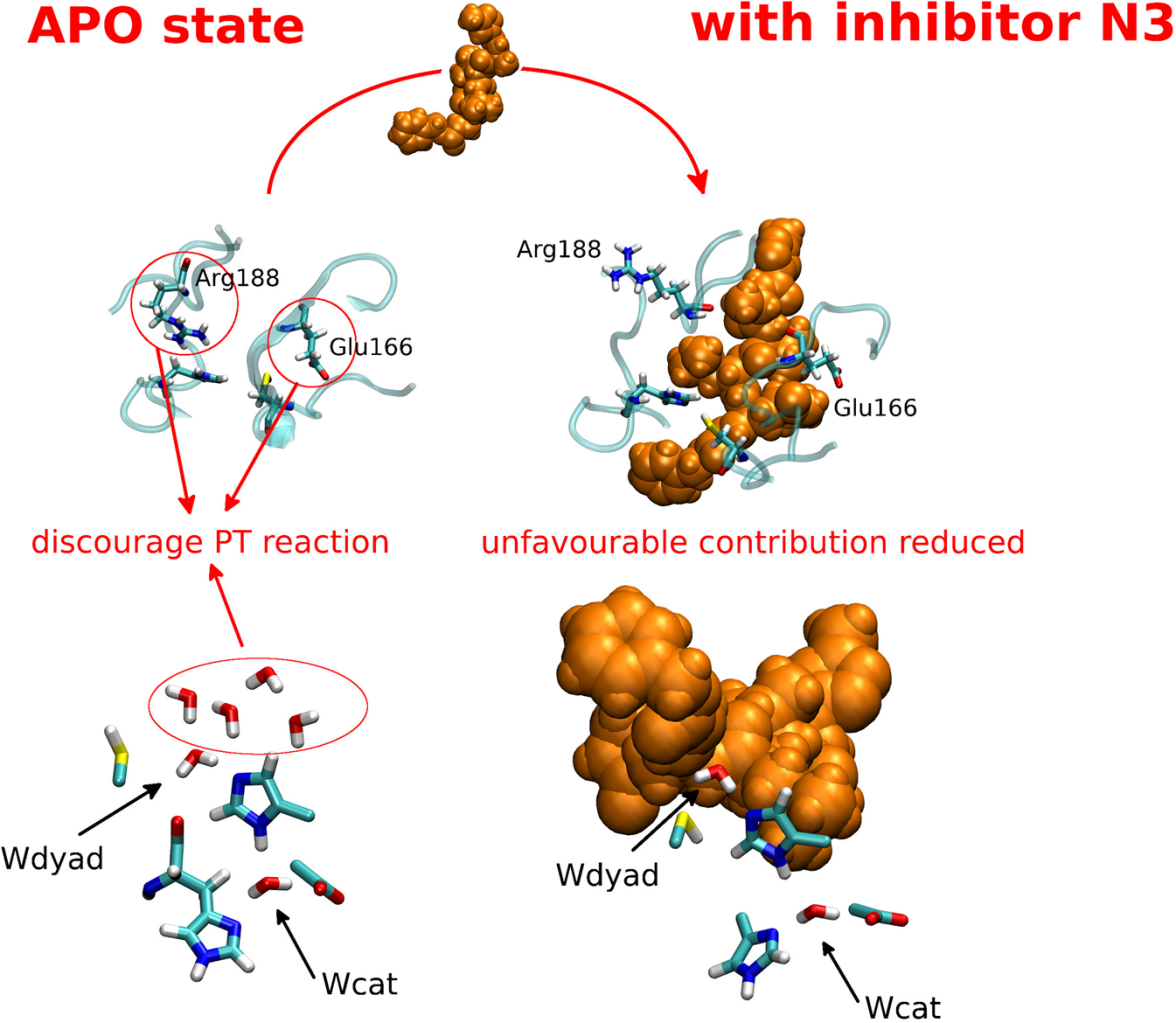
Schematic representation of the mechanism that determines a lower PT free energy in the presence of the N3 inhibitor. In the upper panels the positions of the side chains of Arg188 and Glu166 are highlighted in the apo and N3-bound systems. In the bottom panels the positions of the water molecules closest to the catalytic dyad, including the two highly conserved water molecules Wcat and Wdyad, are highlighted in the apo and N3-bound systems. While Wcat and Wdyad are maintained in the N3-bound state, the small water wires in close proximity to Wdyad are expelled upon binding of the inhibitor.

The analysis of the occupancy of water molecules close to the catalytic dyad (i.e., the water molecules residing within 0.5 nm of any atom of the dyad) along the apo-state simulation reveals that, besides the two aforementioned conserved water molecules, there is a high occupancy of small water wires of 3–4 molecules starting from Wdyad (see Figure 4). Therefore, the contributions of Wcat, Wdyad, and the small water wires identified were analyzed separately in the PT energetics analysis (see Figure 3B). It can be seen that there is an opposite effect between Wcat (negative contribution favoring the PT reaction), and Wdyad and the small water wires (positive contribution disfavoring the PT reaction) that almost counterbalance, giving rise to an almost null total contribution of the solvent.

The analysis of the residues and water molecules that contribute the most to the PT energy was also performed for the MD simulation in the reactant ensemble in the presence of N3, along with the direct contribution of N3. The results, reported in Figure 3C, show that in the presence of the inhibitor the residues that most significantly contribute to the PT energy are those already identified in the apo state. In Figure 3E the difference Δ(*qV*) between the single residue contribution obtained from the MD in the presence of N3 and that obtained in the apo (Δ(*qV*) = *qV*(N3) – *qV*(apo)) is also reported. This difference highlights the protein regions that more relevantly contribute to the variation of the energy change upon PT in the presence of N3: the residues with a high negative contribution in Figure 3E are those that contribute to lowering the PT energy in the presence of the inhibitor with respect to the apo state.

The most relevant (positive and negative) contributions are exerted by Glu166, Arg188, N3, and the solvent. Concerning Glu166 and Arg188, the analysis of the crystal structure of SARS-CoV-2 M^pro^ bound to N3 highlights that the inhibitor backbone forms an antiparallel sheet with residues 164–168 on one side and residues 189–191 on the other side. These interactions are essentially preserved in the MD simulations of the non-covalent complex (see SI, Figure S6). The local interactions of N3 with protein sites different from the catalytic site thus favor the PT reaction. The presence of N3 reduces the electrostatic effect of Arg188 and Glu166 that disfavors the PT in the apo state, both by screening the catalytic dyad from their charge and by sterically pushing away their side chains (see Figure 4). This reduced electrostatic effect contributes to the observed lowering of the PT energy.

Concerning the direct effect of the presence of N3, it can be seen that the electrostatic contribution of N3 in Figure 3C is positive and therefore does not favor the lowering of the PT energy. It should be, however, noted that this contribution only takes into account the direct electrostatic effect of N3, neglecting any possible higher-level interaction between the inhibitor and the catalytic dyad. The positive contribution of N3 is counterbalanced by a negative contribution of the solvent, which implies that the desolvation of the active site occurring upon binding of N3 gives a favorable contribution to the PT process. Although this result might sound counterintuitive (i.e., that PT is favored by desolvation), it is very well explained by the detailed analysis of the contributions of the water molecules residing closest to the catalytic dyad, as described hereafter.

The contribution of Wcat in the N3-bound state is essentially the same as that in the apo state (see Figure 3, panels B and D), consistent with the fact that in the presence of the inhibitor the same HB pattern for Wcat is observed as the apo state (see Figure 4 and Figure S9). Preservation of this HB pattern was already observed in a previous simulation of a non-covalent complex formed between the enzyme and a model peptide mimicking the polyprotein sequence recognized at the active site.^41^ In contrast, significant differences are observed in the contributions of Wdyad and its water wires, that are less positive (i.e., more favorable to PT) than in the apo state (see Figure 3, panels B and D). These changes in the electrostatic interactions exerted on the catalytic dyad are related to differences in the structural organization of the water molecules. In fact, Wdyad is less stable in the presence of the inhibitor (its occupancy is ∼80% in the apo state and ∼55% with N3), and the relative orientation of Wdyad with respect to the catalytic dyad changes. In addition, the water wires are expelled upon binding of the inhibitor (representative configurations showing the different arrangement of these water molecules are reported in Figure 4). In other words, the small water wires, which have an unfavorable electrostatic contribution to the PT in the apo state, are released upon binding of the inhibitor, thereby making the PT more favorable. As a last remark, in the presence of N3 the feasibility of the PT reaction is also enhanced by a better relative position of the two reaction partners (Cys145 and His41). In fact, the distance between the sulfur atom of Cys145 and the N_ε_ atom of His41 is lower and less variable in the MD simulation with N3 than in the apo-state simulation (see Figure S7). This result is in agreement with what was observed in previous simulations, wherein the apo state and a non-covalent complex between the enzyme and a model peptide mimicking the polyprotein sequence recognized at the active site were investigated and compared.^41^

Covalent inhibition in enzymes is a rather complex process, involving both thermodynamics and kinetics and influenced by multiple elements, such as solvent effects and conformational fluctuations.^33,42^ Thus, the thermodynamic feasibility of the PT reaction is not the only factor influencing the reactivity of covalent inhibitors. Nonetheless, the approach presented in this work is envisioned to be of significant aid in the design of new inhibitors by means of computer simulations. In particular, our approach can help identify compounds that can promote the catalytic PT reaction and, therefore, be good candidates as covalent inhibitors, complementing the information that can be obtained from the available computational approaches for drug design.^43−47^ Knowledge of the key residues and water molecules that tune the catalytic PT reaction can in fact guide the analysis of the interactions between the recognition moieties of a candidate compound and the different sub-sites of the binding pocket of the protein, which is a crucial step in the design and screening of potential inhibitors. In addition, identification of specific conserved water molecules able to affect the PT energetics could be used in docking procedures to select which water molecules should be explicitly considered. More generally, the present approach can be used to reveal key sites in enzymes that can be mutated in order to affect the catalytic reaction. In addition, the same sites can be targeted with ligands, also in the framework of allosteric inhibition, to suppress the enzymatic activity.

## Methods

The theoretical background of the MD-PMM approach is described in the SI. In what follows, we outline the calculations performed to obtain the PT free energy.

1. In the MD-PMM approach, the first step consists in defining the portion of the system responsible for the chemical event (in the case of PT, the donor/acceptor sites) to be treated at the quantum-mechanical level. In the present case, the side chains of Cys145 and His41 were selected either as a single or as two separate QCs (see above).
2. MD simulations of the whole quantum-center/external-environment system were performed to sample the configurational space of the system.
3. Quantum-mechanical calculations were performed on the isolated QC(s) at the DFT level to obtain the unperturbed Hamiltonian of the QC(s).
4. For each MD configuration of the whole system, the perturbing effect of the external environment was evaluated: the electrostatic potential and electric field exerted on the QC center of mass by each atom belonging to the environment was calculated.
5. For each MD configuration, the perturbed Hamiltonian (depending on the instantaneous values of the electrostatic potential and electric field) was constructed and diagonalized to provide a set of eigenvalues and eigenvectors.
6. From the eigenvalues and eigenvectors of the perturbed Hamiltonian, the QC properties/observables of interest in the presence of the perturbing environment can be calculated as a function of time. In the present case, the energy change upon PT was evaluated for each MD configuration. From this energy time series the free energy change upon PT was calculated.

## Supporting Information Available

SARS-CoV-2 M^pro^ structure; details on the MD-PMM approach; details on the quantum mechanical calculations; details on the molecular dynamics simulations; additional structural analyses; comparison of the His41E apo state with the 13b-bound state (PDF)
