# Supplementary material for: Tuning Proton Transfer Thermodynamics in SARS-CoV-2 Main Protease: Implications for Catalysis and Inhibitor Design
Source: J Phys Chem Lett. 2021 Apr 26;12(17):4195–202. doi: 10.1021/acs.jpclett.1c00425 (PMC8097931; doi:10.1021/acs.jpclett.1c00425)
Supplement: Supplementary file 1 — jz1c00425_si_001.pdf [file jz1c00425_si_001.pdf]

Supporting Information: Tuning Proton Transfer Thermodynamics in SARS-Cov-2 Main Protease: Implications for Catalysis and Inhibitors Design

Laura Zanetti-Polzi,[*,†] Micholas Dean Smith,[‡] Chris Chipot,[¶,§] James C. Gumbart,[‖] Diane L. Lynch,[‖] Anna Pavlova,[‖] Jeremy C. Smith,[⊥,‡] and Isabella Daidone[*,#]

†*Center S3, CNR Institute of Nanoscience, Via Campi 213/A, I-41125 Modena, Italy*

‡*Department of Biochemistry, Molecular and Cellular Biology, The University of Tennessee, Knoxville. 309 Ken and Blaire Mossman Bldg. 1311 Cumberland Avenue, Knoxville, TN 37996, United States*

¶*UMR 7019, Universite de Lorraine, Laboratoire International Associe CNRS*

§*University of Illinois at Urbana-Champaign, 1110 West Green Street, Urbana, IL, 61801, United States*

‖*School of Physics, Georgia Institute of Technology, Atlanta GA 30332, United States*

⊥*UT/ORNL Center for Molecular Biophysics, Biosciences Division, Oak Ridge National Laboratory, TN 37831, United States*

#*Department of Physical and Chemical Sciences, University of L'Aquila, Via Vetoio, I-67010 L'Aquila, Italy*

E-mail: laura.zanettipolzi@nano.cnr.it; isabella.daidone@univaq.it

SARS-CoV-2 M^{pro} structure

SARS-CoV-2 M^{pro} is a three-domain cysteine protease. Domains I (residues 8-101) and II (residues 102-184) are arranged in an antiparallel β-barrel structure, whereas domain III (residues 201-303) contains five α-helices arranged in a globular cluster.[S1] Domain III has been suggested to be essential in the proteolytic activity by keeping domain II and the long loop connecting domains II and III (residues 185-200) in the proper orientation, and/or by orienting the N-terminal residues that are essential for the dimerization.[S2] Dimerization of the enzyme was itself shown to be essential for catalytic activity by maintaining the proper shape of the pocket of the substrate-binding site.[S3] Among the interactions relevantly affecting the catalytic dyad residues, Cys145 and His41, the hydrogen bond between Cys145 backbone oxygen and Asn28 side chain nitrogen, that is present in the crystal structure, is suggested to be involved in the orientation and flexibility of the C-terminal portion of the oxyanion loop. In agreement with previous simulations,[S4] this interaction is stable along the MD simulation with His41D in the apo state (Figure S1). Concerning His41, a salt bridge interaction between Arg40 and Asp187 contributes to maintain in the right place the small helix containing the catalytic histidine and the first part of the loop connecting domains I and II. Also in agreement with previous MD simulations,[S4] this interaction, that is present in the crystal structure, is maintained in the MD simulation with His41D in the apo state (Figure S2).

The residues that most relevantly contribute to the proton transfer (PT) energy (see main text) are Arg40, Glu166 and Arg188 (positive contributions disfavouring PT) and Asp187 (negative contribution favouring PT). These are charged residues in the vicinity of the catalytic dyad that, besides being energetically relevant, are also structurally relevant. Arg40 and Asp187 are bound in a salt bridge (see above). Glu166 forms a salt bridge with the amino group of the N-terminus residue of the other monomer (Ser1) and is involved in the dimerization.[S3] In addition, the native loop containing Asp187 and Arg188 was shown to be essential for the proteolytic activity of a very similar protease from another coronavirus.[S5]

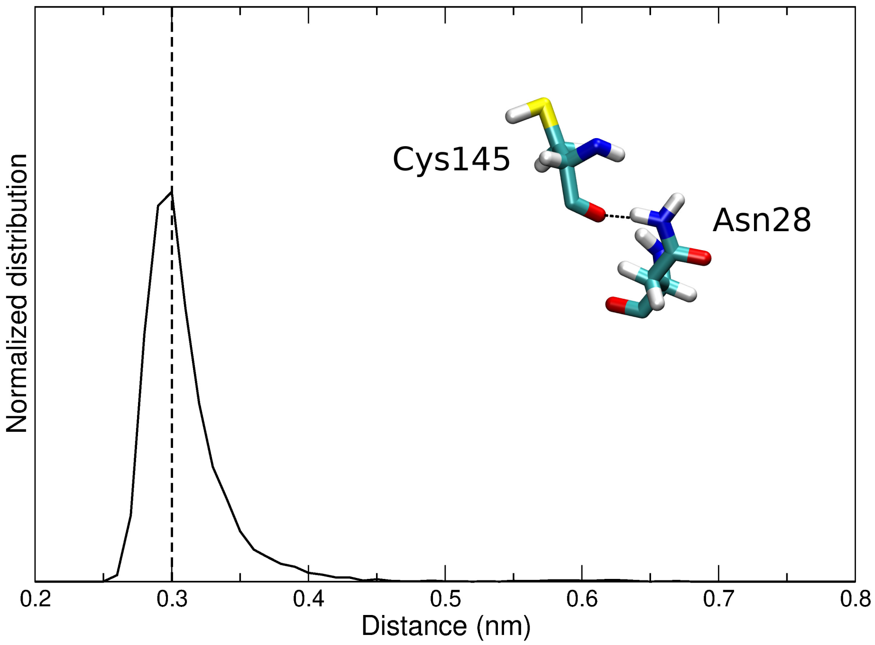

Figure S1: Normalized distribution of the distance between the backbone oxygen of Cys145 and the side chain N_δ of Asn28 in the MD simulation with His41D in the apo state. The vertical line shows the corresponding distance in the crystal structure.

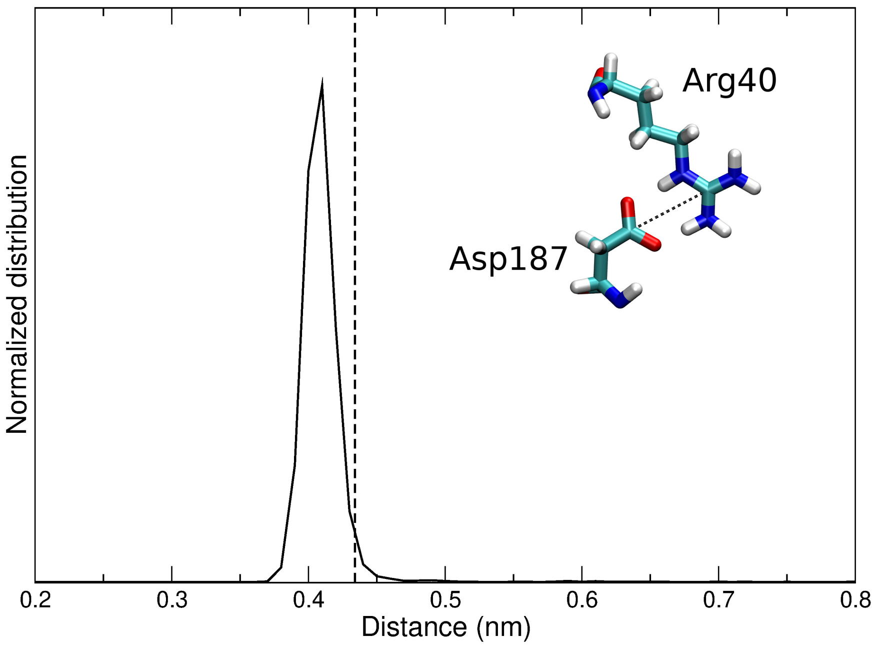

Figure S2: Normalized distribution of the distance between the guanidine carbon of Arg40 and the gamma carbon of Asp187 in the MD simulation with His41D in the apo state. The vertical line shows the corresponding distance in the crystal structure.

The Perturbed Matrix Method

The MD-PMM approach is a hybrid quantum/classical theoretical-computational approach based on molecular dynamics (MD) simulations and on the pertubed matrix method (PMM.[S6,S7] As commonly done in hybrid multiscale approaches,[S8–S11] also for the investigation of enzyme catalysis,[S12–S18] the portion of the system in which the chemical event takes place is treated quantum mechanically (the quantum center, QC) while the rest of the system is treated classically and atomistically and exerts an electrostatic perturbation on the QC electronic states. The main difference with other hybrid methods is that in the MD-PMM, the whole system (including the QC) phase space is sampled by classical MD simulations, allowing an extensive sampling of the QC and environment configurational space. The electrostatic perturbation of the environment is included *a posteriori*: the electronic properties of the isolated QC (unperturbed properties) are calculated quantum-chemically in vacuum (i.e., in the gas phase) and then, for each configuration generated by all-atoms classical MD simulations of the whole system, the electrostatic effect of the instantaneous atomistic configurations of the environment is included as a perturbing term within the QC Hamiltonian operator. This allows to take into account the effect of the fluctuating perturbing environment (the solvent and the part of the solute which is not treated at the quantum level) on the quantum properties of the QC.

For each configuration of the whole system obtained from the MD simulation, the effect of the external environment on the QC eigenstates is included by building and diagonalizing the perturbed electronic Hamiltonian matrix $\widehat{H}$ constructed in the basis set of the unperturbed Hamiltonian eigenstates of the QC. Indicating with $\mathcal{V}$ and $\mathbf{E}$ the perturbing electric potential and field, respectively, exerted by the environment on the QC:

$$\widehat{H} \cong \widehat{H}^0 + \widetilde{I} q_T \mathcal{V} + \widetilde{Z}_1 \tag{1}$$

$$[\widetilde{Z}_1]_{j,j'} = -\mathbf{E} \cdot \langle \phi_j^0 | \hat{\boldsymbol{\mu}} | \phi_{j'}^0 \rangle \tag{2}$$

where $\widehat{H}^0$ is the QC unperturbed electronic Hamiltonian (i.e., as-obtained considering the isolated QC) and q_T, $\hat{\boldsymbol{\mu}}$ and ϕ_j^0 are the QC total charge, dipole operator and unperturbed electronic eigenfunctions, respectively, $\widetilde{I}$ is the identity matrix and the angled brackets indicate integration over the electronic coordinates.

At each frame of the MD simulation, the perturbed electronic Hamiltonian matrix is constructed and diagonalized, providing a continuous trajectory of perturbed eigenvalues and eigenvectors to be used for evaluating the QC instantaneous perturbed quantum observable of interest as, in the present case, the QC ground state energy in the protonated and deprotonated states. More details on the method can be found in the original articles.[S6,S19]

For the more specific task of investigating the proton transfer thermodynamics, the free energy change associated to the PT reaction between Cys145 and His41 is calculated. Given the PT reaction:

$$\text{Cys145H} + \text{His41} \rightleftharpoons \text{Cys145}^- + \text{His41H}^+ \tag{3}$$

the energy variation upon PT (the PT energy) is calculated with the MD-PMM approach for each configuration obtained from the MD simulations in both the reactant and the product states as defined in Eq. 3.

In the MD simulations, Cys145 and His41 sample a highly variable range of relative conformations, including configurations in which a direct (hydrogen bond) HB between the sulfur of Cys145 and the ε nitrogen of His41 involving the proton to be exchanged is present. In the absence of a direct HB between the catalytic dyad residues, Cys145 and His41 are treated as separate QCs in the MD-PMM, while for the configurations in which this HB is present, Cys145 and His41 are treated as a unique QC.

When treated separately, the two QCs interact with each other as well as with their en-

vironment. The perturbed states of Cys145, either in the protonated or in the deprotonated condition, are obtained considering the corresponding QC as perturbed by the electric field provided by His41 as well as the rest of the atomic-molecular system, both treated within the semiclassical approximation. The same procedure is performed for His41, the corresponding QC of which is perturbed by Cys145 and the rest of the atomic-molecular system treated within the semiclassical approximation. The diagonalization at each MD frame of the perturbed electronic Hamiltonian matrices (see Eq. 1) with either Cys145 or His41 (in the protonated and in the deprotonated condition) allows the calculation of the perturbed energy variation upon PT at each MD configuration i.e., the time evolution of the PT energy $\Delta\varepsilon_{sep}$. To improve the approximation of treating the donor and acceptor as separate QCs, we consider two possible reaction paths for the PT (see Eq. 4). The deprotonation energy of Cys145 is calculated by including either protonated or deprotonated His41 in the perturbing environment. Analogously, the protonation energy of His41 is calculated by including either protonated or deprotonated Cys145 in the perturbing environment. $\Delta\varepsilon_{sep}$ is then calculated by averaging at each MD frame the time evolution of the PT energy obtained from the two reaction paths reported in the following reaction equation:

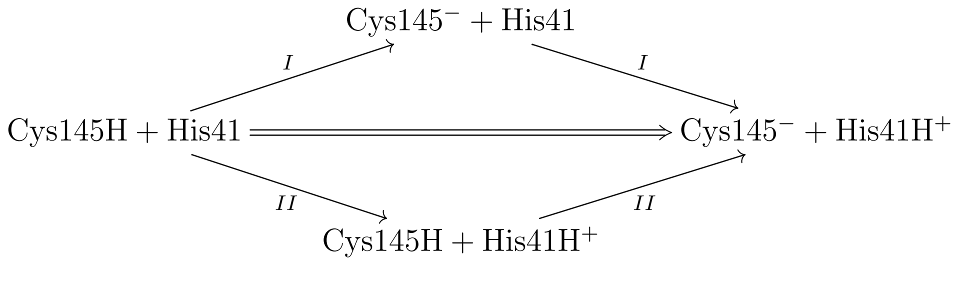

(4)

In the configurations in which a direct Cys145-His41 HB is formed, and therefore a single QC is considered, the relative orientation of the two residues is scarcely variable. The most probable relative orientation of Cys145 and His41, as provided by each simulation condition, is used to perform QM calculations of the complex, with the proton either on Cys145 or on His41. The perturbed states of the complex, in both protonation states, are then obtained

considering the single QC as perturbed by the electric field provided by the rest of the atomic-molecular system, treated within the semiclassical approximation. This again allows the calculation of the perturbed energy variation upon PT at each MD configuration i.e., the time evolution of the PT energy $\Delta\varepsilon_{sing}$.

The final time evolution of the PT energy $\Delta\varepsilon$ is then obtained by considering $\Delta\varepsilon_{sep}$ for the configurations in which no direct Cys145-His41 HB is present, and $\Delta\varepsilon_{sing}$ for the configurations in which the HB is present.

Then, the Gibbs free energy change ΔG^0 associated to the PT can be calculated as:

$$\begin{aligned} \Delta G^0 &= -k_B T \ln\langle e^{-\beta\Delta\mathcal{H}}\rangle_R = k_B T \ln\langle e^{\beta\Delta\mathcal{H}}\rangle_P \\ &\cong -k_B T \ln\langle e^{-\beta\Delta\varepsilon}\rangle_R = k_B T \ln\langle e^{\beta\Delta\varepsilon}\rangle_P \end{aligned} \tag{5}$$

In the above equation $\Delta\mathcal{H}$ is the QC-environment whole energy change upon PT, with $\Delta\varepsilon$ the corresponding QC perturbed electronic ground state energy change. The angle brackets subscripts R and P indicate that both the energy change as well as the averaging are obtained either in the reactant (R) or product (P) ensemble as defined in Eq. 3, and the approximation $\Delta\mathcal{H} \cong \Delta\varepsilon$ is used, i.e., the environment internal energy change associated with the QC reaction is disregarded (being exactly zero when considering typical MD force fields).

From Eq. 5 it follows that $-k_B T \ln\langle e^{-\beta\Delta\varepsilon}\rangle_R$ and $k_B T \ln\langle e^{\beta\Delta\varepsilon}\rangle_P$ provide the upper and lower bounds of ΔG^0 and hence it can be written:

$$\Delta G^0 \cong \frac{k_B T}{2} \ln \frac{\langle e^{\beta\Delta\varepsilon}\rangle_P}{\langle e^{-\beta\Delta\varepsilon}\rangle_R} \tag{6}$$

In this last equation the perturbed electronic ground state energy change as well as the ensemble averages are evaluated via the previously described MD-PMM approach, i.e. by diagonalizing at each MD frame the perturbed electronic Hamiltonian matrices (Eq. 1).

As a further test, we compared the free energy estimated obtained by using the above outlined procedure (i.e. a single QC when the Cys145-His41 HB is present and two separate QCs when this HB is not present) to the one estimated by using two separate QCs for all the frames of the MD simulations. We obtain from the two approaches identical results within the estimated errors.

The PT reaction free energy was also calculated within the linear response approximation, i.e. by assuming a Gaussian distribution for the energy change upon PT and considering the average of the mean values of the PT energy obtained in the two ensembles along the MD trajectories.[S20,S21] The results are in qualitative agreement with those obtained by explicitly calculating the reaction free energy ΔG^0 (see Eq. 6) providing estimates for the free energy change upon PT of 40 and 49 kJ/mol for His41D and His41E, respectively, with a standard error of $\approx$ 5 kJ/mol. The full calculation of ΔG^0, being based on an exact relation (see Eq. 6), provides a more accurate result than the one that can be obtained within the linear response approximation. However, the latter is less affected by inaccuracies due to finite-sampling issues. The qualitative agreement between the results obtained with the two approaches enhances therefore the reliability of the computed estimates.

For the calculation of the PT reaction free energy in the presence of the inhibitor N3 we did not use the full free energy calculation (with Eq. 6) because the configurational sampling of the non-covalent complex between the protein and the inhibitor (100 ns) was not as extended as for the apo system (almost 1 μs). As a reliable estimate of the free energy with Eq. 6 requires a very extended sampling, in the presence of N3 we use the energy change upon PT to estimate the reaction free energy, i.e. we use the linear response approximation,[S20] that is less affected by inaccuracies due to sampling problems. Then, to improve the free energy estimate, we shifted the computed values obtained in the presence of N3 within the linear response approximation by the same energy difference that is obtained in the apo state between the linear response approximation and the full free energy calculation with Eq. 6 (i.e., by -9 kJ/mol, see above). This shift takes into account the deviations from

the linear response approximation due to the instantaneous fluctuations of the PT energy and to the reorganization energies in the reactant and product ensemble,[S21] which can be reasonably assumed approximately equal in the apo state and in the presence of the inhibitor.

Quantum mechanical calculations

To calculate the free energy change corresponding to the proton transfer reaction between the Cys145 and His41, both groups are selected as QCs. For Cys145, the side chain group modeled as methanethiol is selected as QC. For His41, the side chain group (imidazole methylated at the C_β site) is selected as QC considering both the N_ε and N_δ protonation states to model His41E and His41D, respectively. For all QCs, quantum chemical calculations are performed in both protonation states (i.e., for Cys145 on methanethiol and methanethiolate, for His41 on the C_β-methylated imidazole, protonated at either N_ε and or N_δ, and C_β-methylated imidazolium) in order to obtain the unperturbed electronic eigenfunctions and properties to be used in the MD-PMM approach. Quantum calculations are performed at the Density Functional Theory (DFT) level[S22] with the 6-31+G(d) basis set[S23] in conjunction with the B3LYP functional[S24] (Time-dependent DFT[S25] is used for evaluating the properties of the excited states). The MD-PMM approach in conjunction with the B3LYP functional provided results in good agreement with the experimental ones for the calculation of the deprotonation free energy in methanethiol (i.e., a cysteine side-chain)[S26] and for the free energy calculation for a PT reaction involving a histidine residue in carbonic anhydrase.[S27] For each protonation state, a 6×6-dimensional Hamiltonian matrix is evaluated and diagonalized at each MD simulation step, according to the MD-PMM procedure (see Perturbed Matrix Method section). All quantum calculations are carried out using the Gaussian09 package.[S28]

Molecular dynamics simulations

To evaluate the proton transfer reaction free energy change, MD simulations are performed in both the reactant and the product states for the active site dyad, i.e, with neutral Cys145 and His41 and in the zwitterionic state (negatively charged deprotonated Cys145 and positively charged double protonated His41). These MD simulations were performed and extensively analyzed in a previouys work.[S29] Hereafter, we recall the simulation protocol and details. For the apo state MD simulations, the structure of the SARS-CoV-2 main protease was taken from the protein data bank, with protein entry 6wqf. The structure was prepared using a combination of the GROMACS[S30,S31] pdb2gmx tool and CHARMM-GUI pdb reader[S32] to set the protonation states of the protein. His164, whose protonation state has been shown to affect the active site stability,[S29] was protonated at its ε nitrogen.

All molecular dynamics simulations were performed using the 2020 version of the GROMACS[S30,S31] software suite with the CHARMM36m force field.[S33] For all systems, the protein was centered in a box with edge-distances of 1 nm and solvated with TIP3P[S34,S35] water along with system charge neutralizing Na+ and Cl- counter-ions. The force-field and run parameters for the MD simulations were obtained from the CHARMM-GUI;[S36] hence, the short-range interactions were treated with a smooth (force-switching) cutoff of 1.2 nm while long-range electrostatics were treated using the Particle-Mesh Ewald (PME) formalism, as implemented in GROMACS.[S37] For all production simulations, a 2-fs integration (MD) timestep was used. To facilitate this timestep, hydrogen-bond distance constraints were restrained with the LINCS algorithm.[S38] Simulations were performed following a three-step approach: minimization, relaxation, and production. During the minimization stage the steepest-descent algorithm (as implemented in GROMACS) was used. Post energy minimization, short (250 ps) NPT relaxation simulations (with default positions restrains generated from CHARMM-GUI) were performed to relax the simulation box dimensions for each replica (at each of the temperature-replica exchange temperature windows, see below). The Berendsen baro/thermostats[S39] was used for the relaxation simulation with integration

time step of 1 fs. The production stage of the simulations make used of a frame saving rate of 10 ps for the non-zwitterionic form and 2 ps for the zwitterionic form. Temperatures and pressures for the production stage were controlled with the V-rescale thermostat[S40] and the Parrinello-Rahman barostat[S41,S42] with the pressure set to 1 bar. The production stage makes use of temperature replica-exchange enhanced sampling (T-REMD).[S43–S46] For the T-REMD, a target exchange probability of 0.2 was selected and a temperature range of 310 K to ≈350 K was chosen to maintain with the number of replicas. To obtain the number and specific temperatures for the replicas, the server developed by Patriksson and van der Spoel[S47] with a target was used. Interestingly, the although a target exchange probability was selected of 0.2, the actual exchange probability was found to be 0.3 for all systems. The zwitterionic form was simulated for 100 ns and the neutral form was simulated for 750 ns.

NAMD 2.13 was used for simulations with the N3 inhibitor.[S48,S49] As in the apo simulations, we used the CHARM36m force field, the TIP3P water model, a 2-fs timestep.[S50,S51] In addition, we used the CGenFF program in order to obtain CHARMM-compatible CGenFF force field parameters for N3.[S52–S54] In order to use a longer 2-fs timestep, all covalent bonds with hydrogens were kept rigid. For long range van der Waals interactions a 1.2 nm cutoff was employed, and a smooth decay to zero was ensured by applying a smoothing function from 1-1.2 nm. For long-range electrostatic interactions the particle-mesh Ewald method was used,[S55] as in the apo simulations. Pressure and temperature were kept constant at biologically relevant values of 1 bar and 310 K using a Langevin thermostat and barostat, respectively.[S56]

The 7BQY[S57] structure was used as a starting point for MD M^{pro} with N3 . Because this structure is missing the C-terminal residues, we used the older N3-bound structure (PDB entry 6LU7) for the C-terminal positions. The structure was solvated and ionized with 0.15 M NaCl in VMD.[S58] After minimization, water and ions were equilibrated for 1 ns while the protein and inhibitor, were restrained with a force constant of 2 kcal/mol/Å^2. In a second equilibration step only the protein backbone was restrained with the same force

constant for 4 ns. Subsequent unrestrained simulations were run in triplicate for 20 ns for each protonation state, and used for the analysis.

Additional structural analyses

In the MD simulations in the apo state reactant ensemble, Cys145 and His41 sample a highly variable range of relative conformations, with an average distance between the sulfur of Cys145 and the ε and δ nitrogen of His41 of ≈0.38 nm and ≈0.50 nm, respectively (see Figure S3). In addition, the side–chain proton of Cys145 only seldom points toward the ε nitrogen of His41 (see Figure S4). In a small number of configurations in the MD trajectory with His41D, the relative position of Cys145 and His41 is however compatible with a direct HB between the sulfur and the ε nitrogen involving the proton to be exchanged (i.e., the sulfur proton). In the MD simulations in the apo–state product ensemble (i.e., the ionic couple) the relative configurations of Cys145 and His41 compatible with a direct S-N_{ε} HB are much more frequent (see Figure S5).

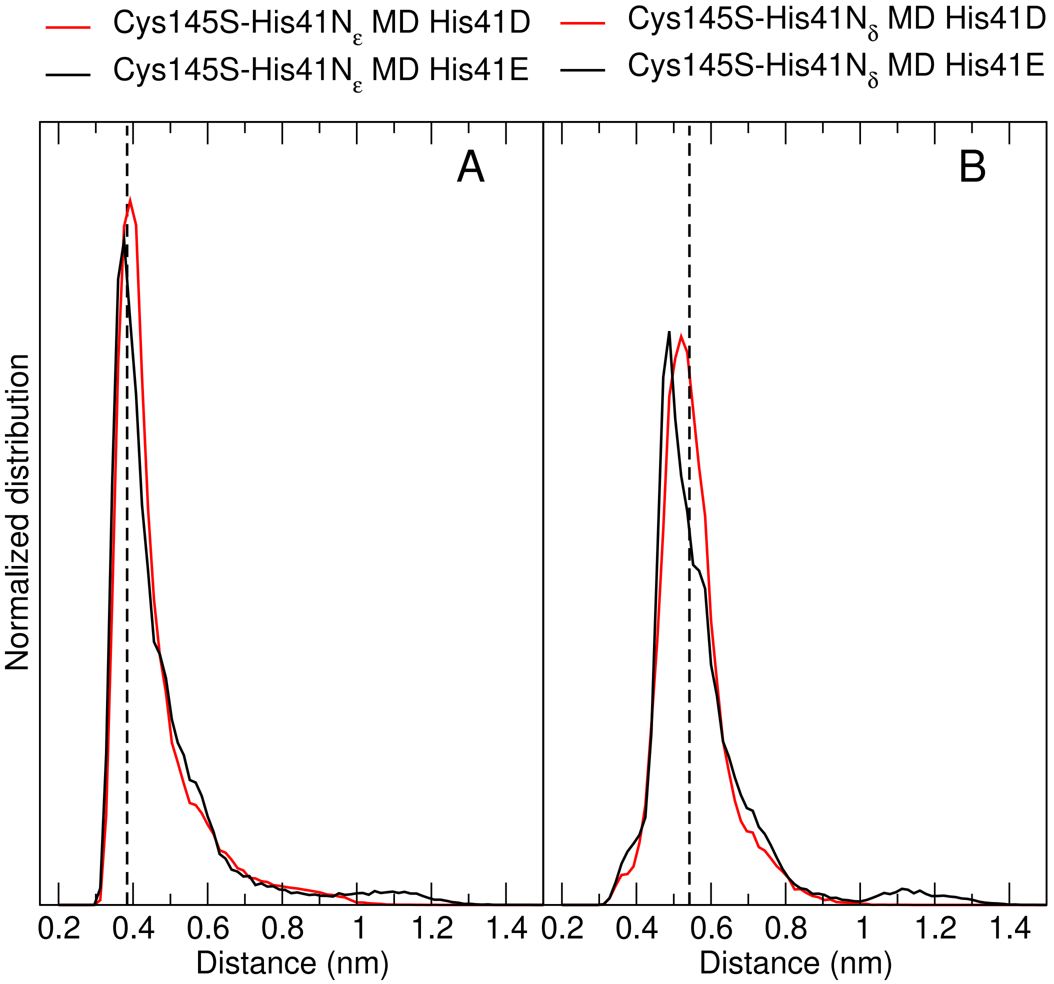

Figure S3: Normalized distribution of the distance between Cys145 S and His41 N_ε (A) and N_δ (B) in the MD simulations with His41E (black) and His41D (red) in the apo state. The vertical lines show the corresponding distances in the crystal structure.

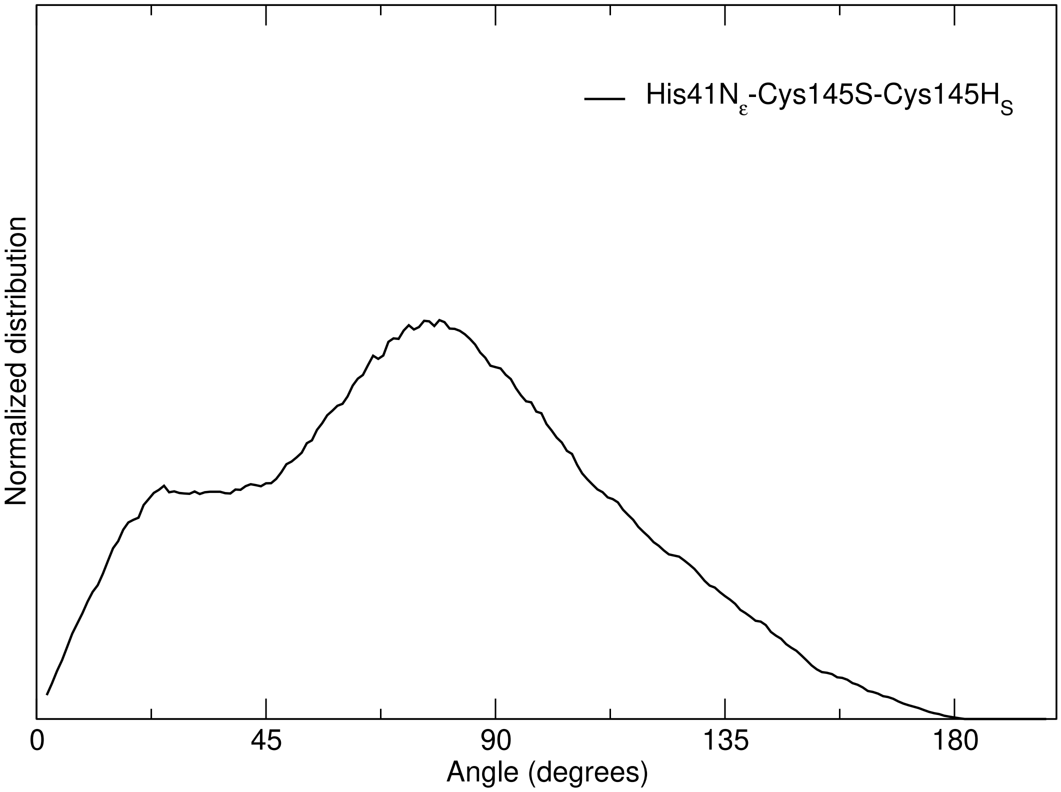

Figure S4: Normalized distribution of the angle His41 N_ε– Cys145 S – Cys145 H_s in the MD simulation with His41D in the apo state (reactant ensemble).

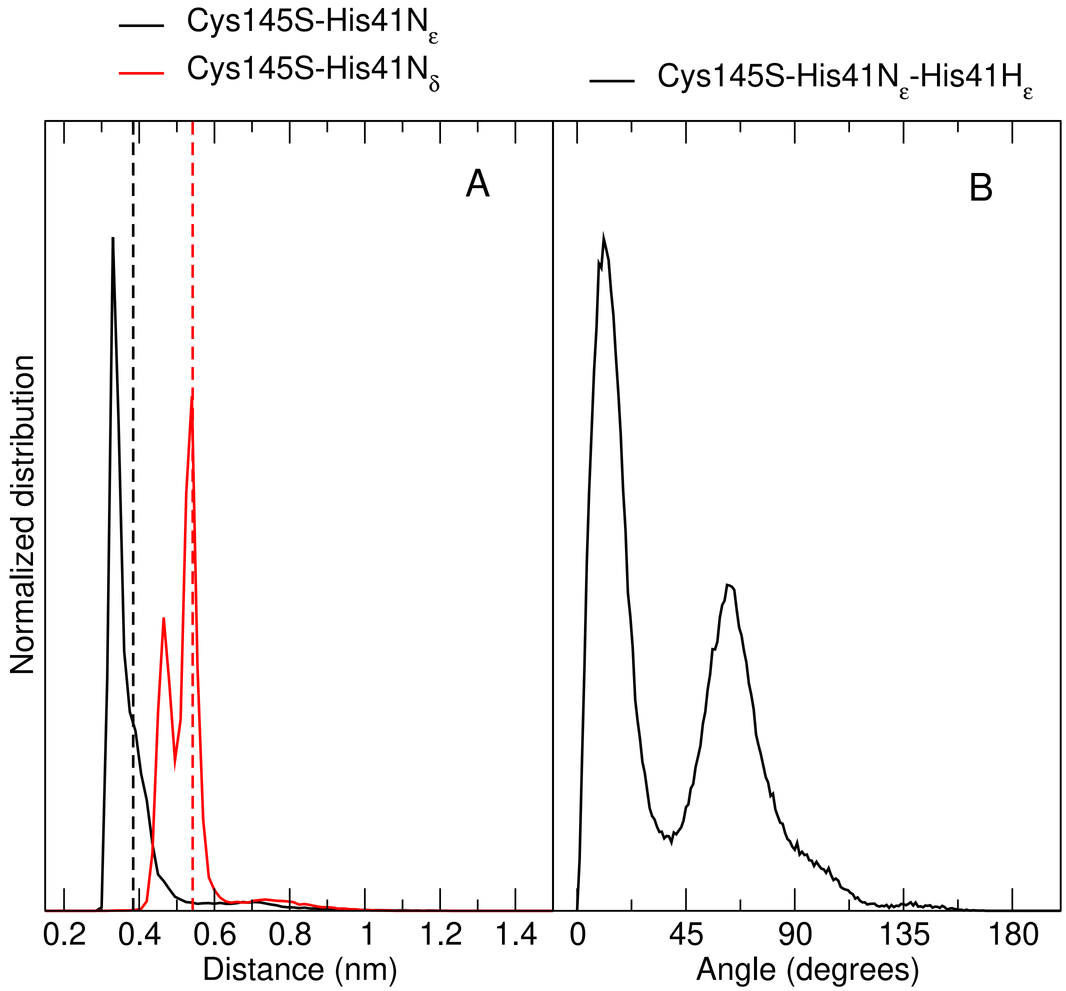

Figure S5: A: Normalized distribution of the distance between Cys145 S and His41 N_ε (black) and N_δ (red) in the MD simulation in the apo state product ensemble. The vertical lines show the corresponding distances in the crystal structure. B: Normalized distribution of the angle Cys145 S – His41 N_ε– His41 H_ε in the MD simulation in the apo state product ensemble.

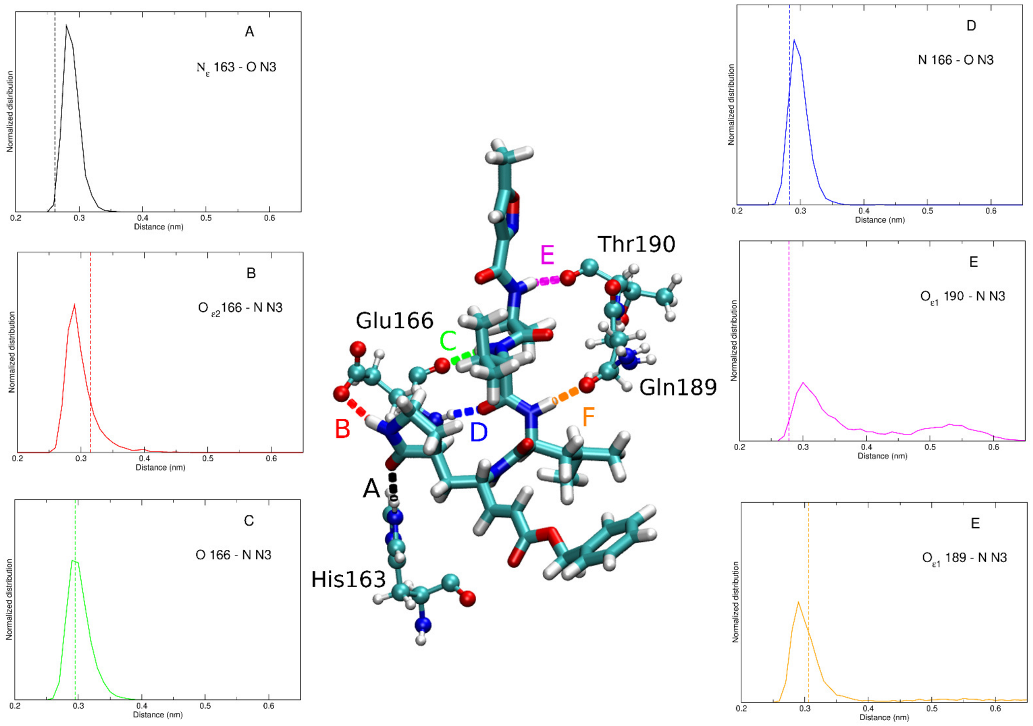

Figure S6: Analysis of the stability of the hydrogen bonding interactions between N3 and the protein in the MD simulation in the reactant ensemble. The normalized distribution of the HB distances is reported and labelled in each panel and in the representative snapshot in the center. The vertical dashed lines show the corresponding distances in the crystal structure. The color code of the curves in each panels is also used to identify the distance in the representative snapshot, in which the inhibitor N3 is reported in licorice and the protein residues in ball and sticks.

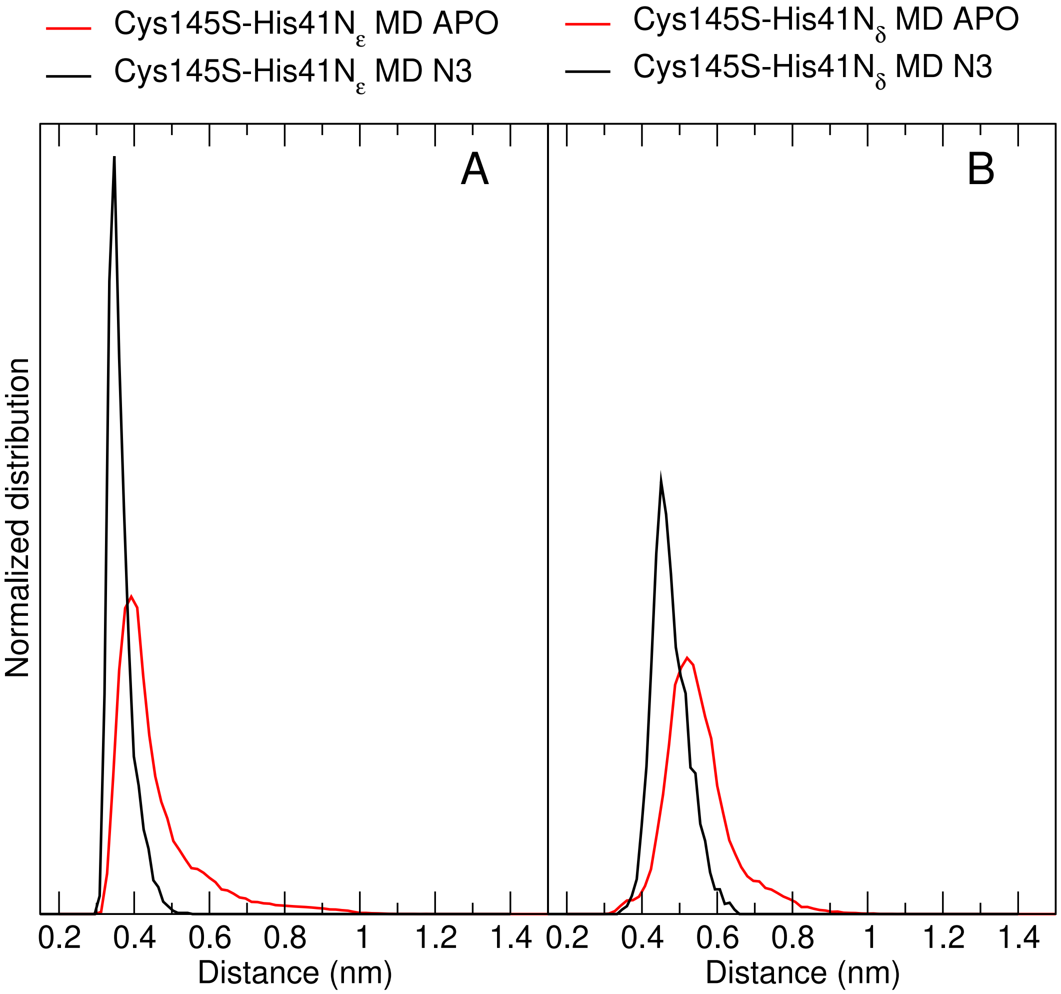

Figure S7: Normalized distribution of the distance between Cys145 S and His41 N_ε (A) and N_δ (B) in the MD simulations in the reactant ensemble in the presence of N3 (black) and in the apo state (red).

Contribution of protein residues and water molecules to the proton transfer energy: the His41E apo state in comparison with the 13b-bound state

As reported in Figure S8C, the residues that most relevantly contribute to the PT energy for the **13b** bound complex are the same that are observed in the apo state (with both His41D, see main text Figure 2A, and His41E, see Figure S8A) and in the presence of N3 (see main text, Figure 2C): Arg40, Asp 48, Glu55, Lys61, Glu166, Asp187, Arg188 and Ser1 of the other monomer. It can be observed, comparing Figure S8A and Figure 2A in the main text, that in the His41D apo state the total contribution of the solvent is almost negligible, while in the His41E apo state the solvent exerts a positive contribution disfavoring the PT reaction.

In Figure S8E the difference Δ(qV) between the single residue contribution obtained from the MD in the presence of **13b** and that obtained in the apo (Δ(qV) = qV (**13b**)-qV (apo)) is also reported, highlighting the protein regions that more relevantly contribute to the variation of the energy change upon PT in the presence of the inhibitor. In Figure S8E the contribution of Glu166 and Arg188, already observed in the presence of N3 and discussed in the main text, can be observed. Two additional relevant contributions from Arg40 and Asp187 can be also observed, which are not present for the N3–bound complex. These contribution arise from a different arrangement of Wcat (*vide infra*) that brings these two residues, that interact *via* a salt bridge, closer to His41. As in the case of the His41D apo state and the N3–bound state, the contributions of Wcat, Wdyad and the small water wires are analyzed separately in the PT energetics analysis (see Figure S8B,D,F).In the **13b**–bound state the contribution of Wcat is essentially the same as in the apo state (see Figure S8, panels B and D). However, this contribution is opposite to the one observed in the His41D apo state (see Figure 2B in the main text). The analysis of the HB network involving Wcat along the MD simulations with His41D and His41E reveals relevant differences. In the His41D

simulation the HB network observed in the crystal structures is essentially maintained in the MD (see Figure S9). In contrast, in the MD simulations with His41E the interaction between Wcat and His41 Nδ is maintained, the one between Wcat and His164 Nδ is only partially maintained and the one with Asp187 side chain oxygens is lost (see Figure S9). In fact, as a consequence of the protonation of His41 at Nε, Wcat changes its orientation, assuming a new configuration that prevents the simultaneous formation of the HBs with Asp187 and His164 (see representative structures in Figure S9). This rotation implies a different electrostatic effect on the side chain of His41, favoring the PT reaction in the His41D reactant state and disfavoring it in the His41E reactant state. The negative peak due to Asp187 and the negative peak due to Arg40 in Figure S8E arise from the fact that in the MD simulation in the presence of **13b**, the interaction between Wcat and His164 is lost while the one with Asp187 is recovered. The protonation of His41 at Nε also determines a different arrangement of Wdyad in the His41E apo state, that differently from what observed in the His41D apo state, slightly favors the PT reaction. A small contribution of the wires can also be observed. In the presence of **13b**, both Wdyad and the wires are expelled form the active site, leading to an overall positive contribution of the solvent.

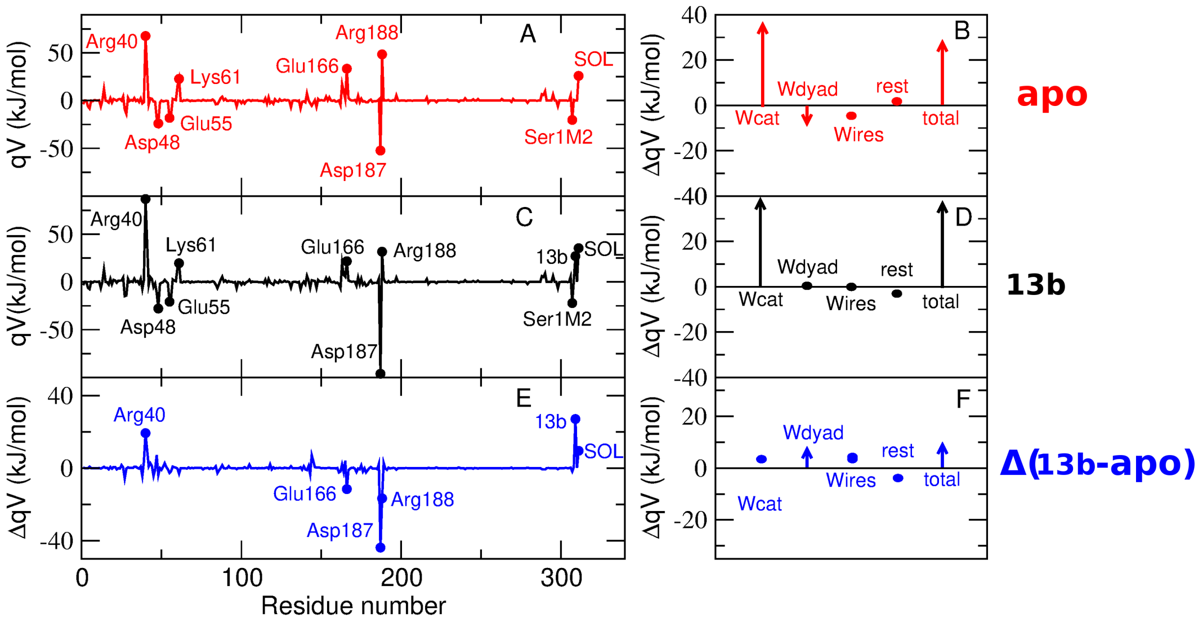

Figure S8: A and C: qV is plotted for each protein residue and all the water molecules as an additional virtual residue SOL for the apo state (A) and in the presence of the inhibitor **13b** (C). qV is the mean value along the MD trajectories of the contribution due to the electrostatic potential to the PT energy. The residues featuring an absolute value of qV higher than 20 kJ/mol are labelled in the figure. The residues with a negative contribution exert an electrostatic effect that favors the PT reaction, while the opposite is true for the residues with a positive contribution. The contributions of the residues of the catalytic dyad (His41 and Cys145) are not included in the plot. E: Δ(qV) = qV(13b)-qV(apo) is plotted for each protein residue and SOL. The residues featuring an absolute value of qV higher than 10 kJ/mol are labelled. The contributions of the residues of the catalytic dyad (His41 and Cys145) are not included in the plot. The residues with a negative contribution are those that contribute to lower the PT energy in the presence of the inhibitor with respect to the apo state while the opposite is true for the residues with a positive contribution. B, D and F: dissection of the contribution of the solvent (SOL): the contribution of Wcat, Wdyad, the molecules forming the wires and the rest of the water molecules are reported together with the total solvent contribution for the apo state (B), in the presence of **13b** (D) and for the difference Δ(qV) = qV(13b)-qV(apo) (F).

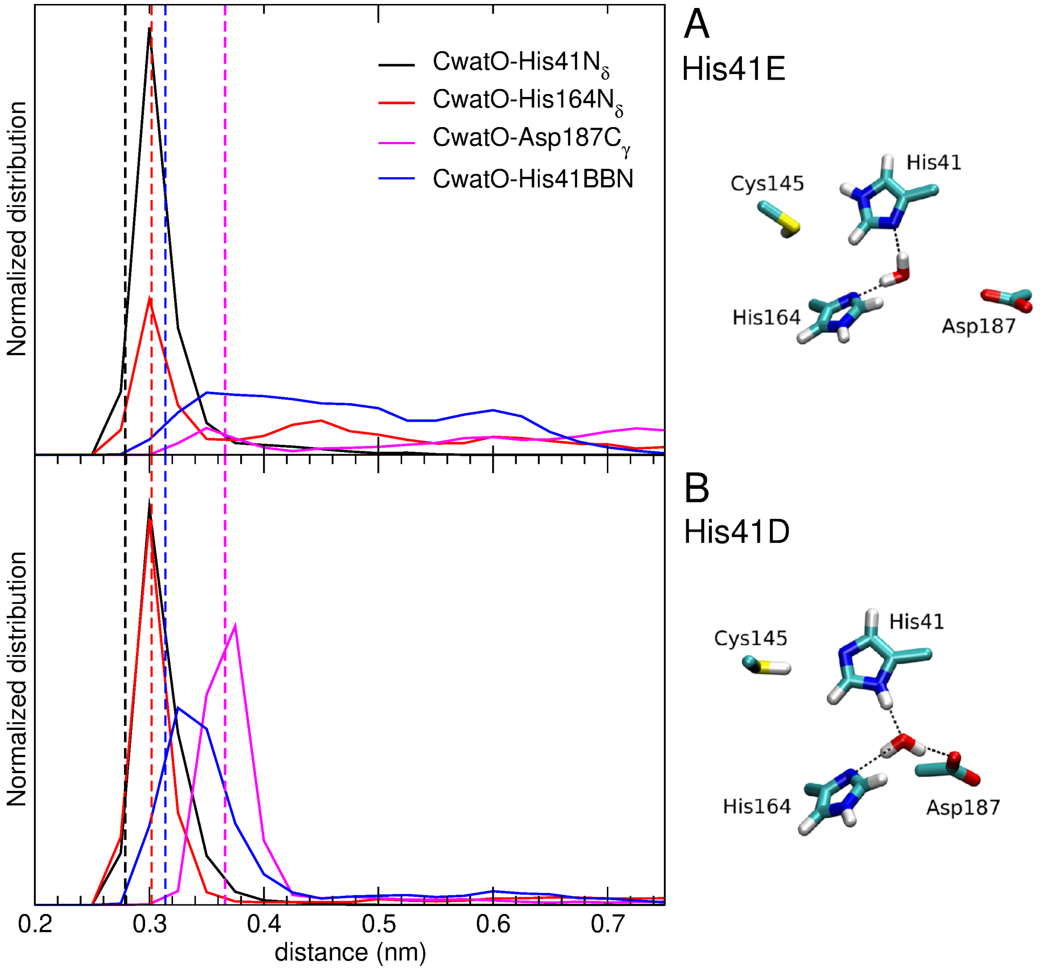

Figure S9: Normalized distribution of the distance between the oxygen atom of Wcat and His41 N_δ (black), His164 N_δ (red), Asp187 C_γ (magenta) and His41 backbone N (blue) in the MD simulation with His41E (A) and His41D (B). The vertical lines show the corresponding distances in the crystal structure. Representative structures of the different hydrogen bonding pattern are also shown.

References

(S1) Jin, Z.; Du, X.; Xu, Y.; Deng, Y.; Liu, M.; Zhao, Y.; Zhang, B.; Li, X.; Zhang, L.; Peng, C.; Duan, Y.; Yu, J.; Wang, L.; Yang, K.; Liu, F.; Jiang, R.; Yang, X.; You, T.; Liu, X.; Yang, X.; Bai, F.; Liu, H.; Liu, X.; Guddat, L. W.; Xu, W.; Xiao, G.; Qin, C.; Shi, Z.; Jiang, H.; Rao, Z. R.; Yang, H. Structure of Mpro from SARS-CoV-2 and discovery of its inhibitors. *Nature* **2020**, *582*, 289–293.

(S2) Yang, H.; Yang, M.; Ding, Y.; Liu, Y.; Lou, Z.; Zhou, Z.; Sun, L.; Mo, L.; Ye, S.; Pang, H.; Gao, G. F.; Anand, K.; Bartlam, M.; Hilgenfeld, R.; Rao, Z. The crystal structures of severe acute respiratory syndrome virus main protease and its complex with an inhibitor. *Proc. Natl. Acad. Sci. USA* **2003**, *100*, 13190–13195.

(S3) Zhang, L.; Lin, D.; Sun, X.; Curth, U.; Drosten, C.; Sauerhering, L.; Becker, S.; Rox, K.; Hilgenfeld, R. Crystal structure of SARS-CoV-2 main protease provides a basis for design of improved α-ketoamide inhibitors. *Science* **2020**, *368*, 409–412.

(S4) Suarez, D.; Diaz, N. SARS-CoV-2 Main Protease: A Molecular Dynamics Study. *J. Chem. Inf. Mod.* **2020**, *60*, 5815–5831.

(S5) Anand, K.; Ziebuhr, J.; Wadhwani, P.; Mesters, J. R.; Hilgenfeld, R. Coronavirus main proteinase (3CLpro) structure: basis for design of anti-SARS drugs. *Science* **2003**, *300*, 1763–1767.

(S6) Aschi, M.; Spezia, R.; Di Nola, A.; Amadei, A. A first priciples method to model perturbed electronic wavefunctions: the effect of an external electric field. *Chem. Phys. Lett.* **2001**, *344*, 374–380.

(S7) Zanetti-Polzi, L.; Del Galdo, S.; Daidone, I.; D'Abramo, M.; Barone, V.; Aschi, M.; Amadei, A. Extending the perturbed matrix method beyond the dipolar approximation: comparison of different levels of theory. *Phys. Chem. Chem. Phys.* **2018**, *20*, 24369–24378.

(S8) Vreven, T.; Morokuma, K. Chapter 3 Hybrid Methods: ONIOM(QM:MM) and QM/MM. *Ann. Rep. Comp. Chem.* **2006**, *2*, 35–51.

(S9) Lin, H.; Truhlar, D. G. QM/MM: what have we learned, where are we, and where do we go from here? *Theor. Chem. Acc.* **2007**, *117*, 185–199.

(S10) Senn, H. M.; Thiel, W. QM/MM methods for biomolecular systems. *Angew. Chem. Int. Ed.* **2009**, *48*, 1198–1229.

(S11) Liu, M.; Wang, Y.; Chen, Y.; Field, M. J.; Gao, J. QM/MM through the 1990s: the first twenty years of method development and applications. *Isr. J. Chem.* **2014**, *54*, 1250–1263.

(S12) Gao, J.; Truhlar, D. G. Quantum mechanical methods for enzyme kinetics. *Ann. Rev. Phys. Chem.* **2002**, *53*, 467–505.

(S13) Sgrignani, J.; Cavalli, A.; Colombo, G.; Magistrato, A. Enzymatic and inhibition mechanism of human aromatase (CYP19A1) enzyme. A computational perspective from QM/MM and classical molecular dynamics simulations. *Mini Rev. Med. Chem.* **2016**, *16*, 1112–1124.

(S14) Faraji, S.; Zhong, D.; Dreuw, A. Characterization of the intermediate in and identification of the repair mechanism of (6-4) photolesions by photolyases. *Angew. Chem. Int. Ed.* **2016**, *55*, 5175–5178.

(S15) Rinaldi, S.; Van der Kamp, M. W.; Ranaghan, K. E.; Mulholland, A. J.; Colombo, G. Understanding complex mechanisms of enzyme reactivity: the case of Limonene-1, 2-epoxide hydrolases. *Acs Catal.* **2018**, *8*, 5698–5707.

(S16) Swiderek, K.; Moliner, V. Revealing the molecular mechanisms of proteolysis of SARS-CoV-2 Mpro by QM/MM computational methods. *Chem. Sci.* **2020**, *11*, 10626–10630.

(S17) Arafet, K.; Serrano-Aparicio, N.; Lodola, A.; Mulholland, A.; González, F. V.; Swiderek, K.; Moliner, V. Mechanism of inhibition of SARS-CoV-2 Mpro by N3 peptidyl Michael acceptor explained by QM/MM simulations and design of new derivatives with tunable chemical reactivity. *Chem. Sci.* **2021**, *12*, 1433–1444.

(S18) Ramos-Guzman, C. A.; Ruiz-Pernia, J. J.; Tunon, I. Unraveling the SARS-CoV-2 main protease mechanism using multiscale methods. *ACS Catal.* **2020**, *10*, 12544–12554.

(S19) Amadei, A.; D'Alessandro, M.; D'Abramo, M.; Aschi, M. Theoretical characterization of electronic states in interacting chemical systems. *J. Chem. Phys.* **2009**, *130*, 08410–08415.

(S20) Muegge, I.; Qi, P.; Wand, A.; Chu, Z.; Warshel, A. The reorganization energy of cytochrome c revisited. *J. Phys. Chem. B* **1997**, *101*, 825–836.

(S21) Amadei, A.; Daidone, I.; Bortolotti, C. A. A general statistical mechanical approach for modeling redox thermodynamics: the reaction and reorganization free energies. *RSC Adv.* **2013**, *3*, 19657–19665.

(S22) Parr, R. G.; Yang, W. Density-functional theory of the electronic structure of molecules. *Ann. Rev. Phys. Chem.* **1995**, *46*, 701–728.

(S23) Krishnan, R.; Binkley, J.; Seeger, R.; Pople, J. Self–consistent molecular orbital methods. XX. A basis set for correlated wave functions. *J. Chem. Phys.* **1980**, *72*, 650–654.

(S24) Becke, A. D. Density-functional thermochemistry. III. The role of exact exchange. *J. Chem. Phys.* **1993**, *98*, 5648–5652.

(S25) Adamo, C.; Jacquemin, D. The calculations of excited-state properties with Time-Dependent Density Functional Theory. *Chem. Soc. Rev.* **2013**, *42*, 845–856.

(S26) Zanetti-Polzi, L.; Daidone, I.; Amadei, A. Fully atomistic multiscale approach for pKa prediction. *J. Phys. Chem. B* **2020**, *124*, 4712–4722.

(S27) Zanetti-Polzi, L.; Aschi, M.; Daidone, I. Cooperative proteinâĂŞsolvent tuning of proton transfer energetics: carbonic anhydrase as a case study. *Phys. Chem. Chem. Phys.* **2020**, *22*, 19975–19981.

(S28) Frisch, M. J.; Trucks, G. W.; Schlegel, H. B.; Scuseria, G. E.; Robb, M. A.; Cheeseman, J. R.; Scalmani, G.; Barone, V.; Mennucci, B.; Petersson, G. A. Gaussian 09, revision A. 1. *Gaussian Inc., Wallingford, CT* **2009**,

(S29) Pavlova, A.; Lynch, D. L.; Daidone, I.; Zanetti-Polzi, L.; Smith, M. D.; Chipot, C.; Kneller, D. W.; Kovalevsky, A.; Coates, L.; Golosov, A. A.; Dickson, C. J.; Velez-Vega, C.; Duca, J. S.; Vermaas, J. V.; Pang, Y. T.; Acharya, A.; Parks, J. M.; Smith, J. C.; Gumbart, J. C. Inhibitor binding influences the protonation states of histidines in SARS-CoV-2 main protease. *Chem. Sci.* **2021**, *12*, 1513–1527.

(S30) Hess, B.; Kutzner, C.; Van Der Spoel, D.; Lindahl, E. GROMACS 4: algorithms for highly efficient, load-balanced, and scalable molecular simulation. *J. Chem. Theory Comput.* **2008**, *4*, 435–447.

(S31) Abraham, M. J.; Murtola, T.; Schulz, R.; Páll, S.; Smith, J. C.; Hess, B.; Lindahl, E. GROMACS: High performance molecular simulations through multi-level parallelism from laptops to supercomputers. *SoftwareX* **2015**, *1*, 19–25.

(S32) Jo, S.; Kim, T.; Iyer, V. G.; Im, W. CHARMM-GUI: a web-based graphical user interface for CHARMM. *J. Comp. Chem.* **2008**, *29*, 1859–1865.

(S33) Huang, J.; Rauscher, S.; Nawrocki, G.; Ran, T.; Feig, M.; de Groot, B. L.; Grubmüller, H.; MacKerell, A. D. CHARMM36m: an improved force field for folded and intrinsically disordered proteins. *Nature methods* **2017**, *14*, 71–73.

(S34) Bjelkmar, P.; Larsson, P.; Cuendet, M. A.; Hess, B.; Lindahl, E. Implementation of the CHARMM force field in GROMACS: analysis of protein stability effects from

correction maps, virtual interaction sites, and water models. *J. Chem. Theory Comput.* **2010**, *6*, 459–466.

(S35) Jorgensen, W. L.; Chandrasekhar, J.; Madura, J. D.; Impey, R. W.; Klein, M. L. Comparison of simple potential functions for simulating liquid water. *J. Chem. Phys.* **1983**, *79*, 926–935.

(S36) Jo, S.; Vargyas, M.; Vasko-Szedlar, J.; Roux, B.; Im, W. PBEQ-Solver for online visualization of electrostatic potential of biomolecules. *Nucleic Acids Res.* **2008**, *36*, W270–W275.

(S37) Abraham, M. J.; Gready, J. E. Optimization of parameters for molecular dynamics simulation using smooth particle-mesh Ewald in GROMACS 4.5. *J. Comp. Chem.* **2011**, *32*, 2031–2040.

(S38) Hess, B. P-LINCS: A parallel linear constraint solver for molecular simulation. *J. Chem. Theory Comput.* **2008**, *4*, 116–122.

(S39) Berendsen, H. J.; Postma, J. v.; van Gunsteren, W. F.; Di Nola, A.; Haak, J. R. Molecular dynamics with coupling to an external bath. *J. Chem. Phys.* **1984**, *81*, 3684–3690.

(S40) Bussi, G.; Donadio, D.; Parrinello, M. Canonical sampling through velocity rescaling. *J. Chem. Phys.* **2007**, *126*, 014101.

(S41) Parrinello, M.; Rahman, A. Polymorphic transitions in single crystals: A new molecular dynamics method. *J. Appl. Phys.* **1981**, *52*, 7182–7190.

(S42) Nosé, S.; Klein, M. Constant pressure molecular dynamics for molecular systems. *Mol. Phys.* **1983**, *50*, 1055–1076.

(S43) Earl, D. J.; Deem, M. W. Parallel tempering: Theory, applications, and new perspectives. *Phys. Chem. Chem. Phys.* **2005**, *7*, 3910–3916.

(S44) Hansmann, U. H. Parallel tempering algorithm for conformational studies of biological molecules. *Chem. Phys. Lett.* **1997**, *281*, 140–150.

(S45) Sugita, Y.; Okamoto, Y. Replica-exchange molecular dynamics method for protein folding. *Chem. Phys. Lett.* **1999**, *314*, 141–151.

(S46) Sugita, Y.; Kitao, A.; Okamoto, Y. Multidimensional replica-exchange method for free-energy calculations. *J. Chem. Phys.* **2000**, *113*, 6042–6051.

(S47) Patriksson, A.; van der Spoel, D. A temperature predictor for parallel tempering simulations. *Phys. Chem. Chem. Phys.* **2008**, *10*, 2073–2077.

(S48) Phillips, J. C.; Braun, R.; Wang, W.; Gumbart, J.; Tajkhorshid, E.; Villa, E.; Chipot, C.; Skeel, R. D.; Kale, L.; Schulten, K. Scalable molecular dynamics with NAMD. *J. Comp. Chem.* **2005**, *26*, 1781–1802.

(S49) Phillips, J. C.; Hardy, D. J.; Maia, J. D. C.; Stone, J. E.; Ribeiro, J. V.; Bernardi, R. C.; Buch, R.; Fiorin, G.; Hénin, J.; Jiang, W.; McGreevy, R.; Melo, M. C. R.; Radak, B. K.; Skeel, R. D.; Singharoy, A.; Wang, Y.; Roux, B.; Aksimentiev, A.; Luthey-Schulten, Z.; Kalé, L. V.; Schulten, K.; Chipot, C.; Tajkhorshid, E. Scalable molecular dynamics on CPU and GPU architectures with NAMD. *J. Chem. Phys.* **2020**, *153*, 044130.

(S50) Jorgensen, W. L.; Chandrasekhar, J.; Madura, J. D.; Impey, R. W.; Klein, M. L. Comparison of simple potential functions for simulating liquid water. *J. Chem. Phys.* **1983**, *79*, 926–935.

(S51) Huang, J.; Rauscher, S.; Nawrocki, G.; Ran, T.; Feig, M.; de Groot, B. L.; Grubmüller, H.; MacKerell, A. D. CHARMM36m: an improved force field for folded and intrinsically disordered proteins. *Nat. Methods* **2017**, *14*, 71–73.

(S52) Vanommeslaeghe, K.; Hatcher, E.; Acharya, C.; Kundu, S.; Zhong, S.; Shim, J.; Darian, E.; Guvench, O.; Lopes, P.; Vorobyov, I.; MacKerell Jr., A. D. CHARMM General Force Field: A Force Field for Drug-Like Molecules Compatible with the CHARMM All-Atom Additive Biological Force Fields. *J. Comput. Chem.* **2010**, *31*, 671–690.

(S53) Vanommeslaeghe, K.; MacKerell, A. D. Automation of the CHARMM General Force Field (CGenFF) I: Bond Perception and Atom Typing. *J. Chem. Inf. Model.* **2012**, *52*, 3144–3154.

(S54) Vanommeslaeghe, K.; Raman, E. P.; MacKerell, A. D. Automation of the CHARMM General Force Field (CGenFF) II: assignment of bonded parameters and partial atomic charges. *J. Chem. Inf. Model.* **2012**, *52*, 3155–3168.

(S55) Darden, T. A.; York, D. M.; Pedersen, L. G. Particle mesh Ewald: An N log N method for Ewald sums in large systems. *J. Chem. Phys* **1993**, *98*, 10089–10092.

(S56) Feller, S. E.; Zhang, Y. H.; Pastor, R. W.; Brooks, B. R. Constant pressure molecular dynamics simulations – The Langevin piston method. *J. Chem. Phys* **1995**, *103*, 4613–4621.

(S57) Jin, Z.; Du, X.; Xu, Y.; Deng, Y.; Liu, M.; Zhao, Y.; Zhang, B.; Li, X.; Zhang, L.; Peng, C.; Duan, Y.; Yu, J.; Wang, L.; Yang, K.; Liu, F.; Jiang, R.; Yang, X.; You, T.; Liu, X.; Yang, X.; Bai, F.; Liu, H.; Liu, X.; Guddat, L. W.; Xu, W.; Xiao, G.; Qin, C.; Shi, Z.; Jiang, H.; Rao, Z.; Yang, H. Structure of M^{pro} from SARS-CoV-2 and discovery of its inhibitors. *Nature* **2020**, *582*, 289–293.

(S58) Humphrey, W.; Dalke, A.; Schulten, K. VMD – Visual Molecular Dynamics. *J. Mol. Graph.* **1996**, *14*, 33–38.
